# Role of the Prefrontal Cortex in Pain Processing

**DOI:** 10.1007/s12035-018-1130-9

**Published:** 2018-06-06

**Authors:** Wei-Yi Ong, Christian S. Stohler, Deron R. Herr

**Affiliations:** 1 Department of Anatomy, National University of Singapore, Singapore, 119260 Singapore; 2 Neurobiology and Ageing Research Programme, National University of Singapore, Singapore, 119260 Singapore; 3 College of Dental Medicine, Columbia University, New York, NY 10032 USA; 4 Department of Pharmacology, National University of Singapore, Singapore, 119260 Singapore

**Keywords:** Prefrontal cortex, Pain, Nociception, Antinociception

## Abstract

The prefrontal cortex (PFC) is not only important in executive functions, but also pain processing. The latter is dependent on its connections to other areas of the cerebral neocortex, hippocampus, periaqueductal gray (PAG), thalamus, amygdala, and basal nuclei. Changes in neurotransmitters, gene expression, glial cells, and neuroinflammation occur in the PFC during acute and chronic pain, that result in alterations to its structure, activity, and connectivity. The medial PFC (mPFC) could serve dual, opposing roles in pain: (1) it mediates antinociceptive effects, due to its connections with other cortical areas, and as the main source of cortical afferents to the PAG for modulation of pain. This is a ‘loop’ where, on one side, a sensory stimulus is transformed into a perceptual signal through high brain processing activity, and perceptual activity is then utilized to control the flow of afferent sensory stimuli at their entrance (dorsal horn) to the CNS. (2) It could induce pain chronification via its corticostriatal projection, possibly depending on the level of dopamine receptor activation (or lack of) in the ventral tegmental area-nucleus accumbens reward pathway. The PFC is involved in biopsychosocial pain management. This includes repetitive transcranial magnetic stimulation, transcranial direct current stimulation, antidepressants, acupuncture, cognitive behavioral therapy, mindfulness, music, exercise, partner support, empathy, meditation, and prayer. Studies demonstrate the role of the PFC during placebo analgesia, and in establishing links between pain and depression, anxiety, and loss of cognition. In particular, losses in PFC grey matter are often reversible after successful treatment of chronic pain.

## Introduction

The prefrontal cortex (PFC) is the region of the cerebral cortex which covers the anterior portion of the frontal lobe. It is situated rostral to Brodmann area 6, including that on the orbital surface, and is particularly well-developed in primates, especially man [1]. The PFC is the key structure that underlies executive functions such as planning, problem solving, and social control. It has the ability to represent information not currently in the environment, and this representational knowledge is used to intelligently guide thought, actions, and emotions, including the inhibition of inappropriate thoughts, distractions, actions, and feelings [2]. The PFC can be divided into the medial prefrontal cortex (mPFC), the orbitofrontal cortex, the ventrolateral PFC, the dorsolateral PFC (DLPFC), and the caudal PFC. The mPFC is composed of granular cortical areas (medial areas 9 and 10) and agranular regions (areas 24, 25, and 32) which encompass the anterior cingulate cortex (ACC, area 24), infralimbic cortex (area 25), and the prelimbic cortex (area 32). The DLPFC is composed of the lateral part of area 9 and all of area 46.

The PFC is important for pain processing. Meta-analysis of studies employing experimental pain stimuli indicates the following brain areas to be positively associated with pain: primary and secondary somatosensory cortices, insular cortex, ACC, PFC, and thalamus [3]. Discrimination of pain intensity activates a ventrally directed pathway, extending bilaterally from the insular cortex to the PFC, while discrimination of the spatial aspects of pain involves a dorsally directed pathway from the posterior parietal cortex to the DLPFC. Both of these tasks activate similar regions of the ACC [4]. Intracranial electroencephalogram (EEG) has identified activation of the posterior insula, operculum, mid-cingulate, and amygdala before conscious activity; activation of the anterior insula, PFC, posterior parietal cortex during time-frames coinciding with conscious voluntary reactions; followed by activation of the hippocampus, perigenual, and perisplenial cingulate cortices well after conscious perception occurred [5].

The anterior insula is connected to the ventrolateral PFC and orbitofrontal cortex, while the posterior insula shows strong connections to the primary somatosensory cortex and secondary somatosensory cortex [6]. The main brain areas that are most consistently activated under painful conditions are the insular cortex and secondary somatosensory cortex, bilaterally. Electrical stimulation of these areas, but not in other candidate brain areas, is able to elicit a painful sensation [7]. In addition, these areas show abnormal bilateral recruitment in response to innocuous stimuli during allodynia and neuropathic pain, possibly as a result of reorganization of thalamocortical inputs, from lateral-posterior to anterior-medial thalamic nuclei [8]. The expression of synaptic GluA1 subunit of the α-amino-3-hydroxy-5-methyl-4-isoxazolepropionic acid (AMPA) receptor is enhanced in the insular cortex after nerve injury, which may contribute to pain sensitization [9].

**Fig. 1:**
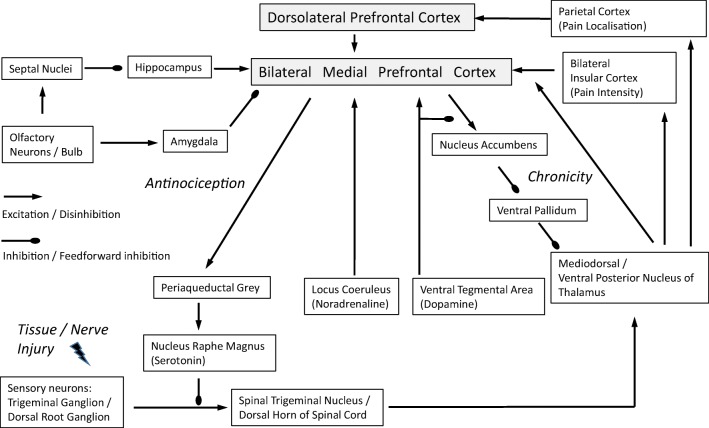
Connections of the PFC related to pain processing

Compared with non-nociceptive stimuli, nociceptive stimuli produce an enhancement of gamma-band oscillations, and these oscillations are a feature of nociceptive signaling in the insula [10] and the mPFC [11]. It has been suggested that increased gamma and theta power in the mPFC, but decreased beta oscillations in the sensorimotor cortex, could be used as biomarkers for pain [12]. The experience of pain can be modified by emotions and expectations [13]. Activation of the mPFC and ACC is related to increased activity of the periaqueductal gray (PAG) [7]. The mPFC is also involved in modulation of pain catastrophizing [14], reduction of pain-induced sympathetic activity [15], and decrease in facial expressions of pain [16] (Fig. 1).

## Neuroanatomy of the PFC Related to Pain

### Connections of the PFC–PAG

The PAG is the primary control center for descending pain modulation and pain relief. It is located around the cerebral aqueduct in the midbrain, and projects to serotoninergic neurons in the nucleus raphe magnus in the rostroventral medulla, which in turn send descending inputs to the spinal trigeminal nucleus in the brainstem or the dorsal horn of the spinal cord for modulation of pain [17]. The cortical projections to the PAG originate principally from the PFC. In particular, this input originates from the mPFC (areas 25, 32), the dorsomedial convexity of the medial wall (area 9) extending into the ACC (area 24), and the posterior orbitofrontal/anterior insular cortex (areas 13, 12) [18]. Microinjections of the neuronal tract tracer, horseradish peroxidase to the PAG, coupled with cortical HRP gel placements in monkeys, provide retrograde and orthograde evidence that the rostral dorsal convexity and rostral mPFC are the principal sources of cortical-PAG projections [19]. Similarly, HRP peroxidase microinjections into different parts of the rostral and caudal PAG in cats provide retrograde evidence that the somatosensory cortex, frontal cortex, insular cortex, and cingulate cortex are significant sources of cortical-PAG projections [20]. In contrast to the mPFC, the lateral part of the frontal lobe (areas 8 and 6) makes a relatively minor contribution, whereas the medial anterior part of the orbitofrontal cortex (areas 11, 13) does not project to the PAG [21]. The DLPFC is, however, connected to the mPFC by short association fibers [22], and it is possible that some of the effects of DLPFC stimulation may be mediated by its connections with the mPFC.

Functional magnetic resonance imaging (fMRI) studies reveal that the ventrolateral-PAG is functionally connected to brain regions associated with descending pain modulation including the ACC, upper pons/medulla, whereas the lateral and dorsolateral subregions of the PAG are connected with brain regions implicated in executive functions, such as the PFC, striatum, and hippocampus [23]. In vivo tracing of neuronal connections using probabilistic tractography seeded in the right DLPFC and left rostral ACC shows that that stronger placebo analgesic responses are associated with increased mean fractional anisotropy values in white matter tracts connecting the PFC with the PAG [24]. In addition, studies using diffusion tensor imaging show that tract paths could be defined between the PFC, amygdala, thalamus, hypothalamus and rostroventral medial medulla bilaterally, and the PAG [25]. The PFC-amygdala-dorsal PAG pathway may mediate fear-conditioned analgesia, i.e., a reduction in pain response upon re-exposure to a context, previously paired with an aversive stimulus [26]. The mPFC-PAG projection also plays a role in modulation of autonomic responses to pain [27] (Fig. 1).

### Connections of the PFC–Thalamic Nuclei

Under physiological conditions, activation of the medial thalamus and increasing the functional connectivity between the thalamus and medial prefrontal cortex has an effect on improving working memory. This could be a mechanism, for example, to convert a painful stimulus into a briefly maintainable construct to guide human behavior and avoid dangerous situations [28]. Under pathological conditions, however, increased thalamocortical input not only to the somatosensory cortex or insula but also the PFC might be a contributing factor in chronic pain. A study using magnetic resonance spectroscopy shows that thalamic inhibitory neurotransmitter γ-aminobutyric acid (GABA) content is significantly reduced in patients with chronic neuropathic pain. This correlates with the degree of functional connectivity between the thalamus and the cerebral cortex, including not only the primary somatosensory cortex, but interestingly, also the secondary somatosensory cortex and anterior insula [29]. These results suggest that increased thalamocortical activity could result in increased activity of the insula and the constant perception of pain.

In animals, pharmacogenetic inhibition of the paraventricular thalamic nucleus attenuates visceral pain induced by pancreatitis. Neurons in the paraventricular nucleus project to cortical layers of the mPFC and synapse with GABAergic inhibitory neurons. Activation of GABAergic neurons occurs during pain, and by contrast, activation of glutamatergic principal neurons in the mPFC reverses visceral nociception [30]. Chronic pain has also been found to induce higher coherence of mediodorsal nucleus of the thalamus–mPFC field potential activities [31]. Results suggest a pronociceptive effect of paraventricular or mediodorsal thalamic inputs, that might occur via activation of GABAergic neurons, and feedforward inhibition of principal neurons in the mPFC that project to the PAG for the suppression of pain. A reduction in thalamocortical input might be relevant in the analgesic effects of gabapentin, a GABA analogue. The latter has been found in animal studies to have an analgesic effect by reversing spared nerve injury (SNI)-induced increases in connectivity between the thalamus and the cortex [32] (Fig. 1).

### Connections of the PFC–Amygdala

The amygdala is involved in fear and fear conditioning, and is reciprocally connected to the PFC [27]. Increased functional connectivity between the left amygdala and multiple cortical, subcortical, and cerebellar regions are found in pain patients, whereas decreased hyperconnectivity between the left amygdala to the motor cortex, parietal lobe, and cingulate cortex is observed after pain rehabilitation treatment [33]. Animal studies show that the basolateral amygdala evokes excitatory and inhibitory responses in cortico-PAG neurons in layer V of both the prelimbic and infralimbic cortices [34]. High-frequency stimulation induces long-lasting suppression of specific high threshold responses of nociceptive neurons in the PFC, and microinjection of NMDA receptor antagonists or metabotropic glutamate receptor (mGluR) antagonists prevent the induction of long-lasting suppression [35]. A selective mGluR1 antagonist also reverses pain-related decrease of background and evoked activity of mPFC neurons in an arthritis pain model [36]. No change in direct excitatory transmission from the amygdala, but an increase in inhibitory transmission is found in the mPFC, in a rat model for arthritic pain [37]. This likely occurs via mGluR1-mediated endogenous activation of GABAA receptors, and could not only result in abnormally enhanced inhibition of principal cells in the mPFC, resulting in reduced output from the mPFC to the PAG and reduced antinociception; but also reduced inhibition of the amygdala itself, possibly contributing to uncontrolled amygdala pain mechanisms [38]. The infralimbic mPFC evokes strong synaptic inhibition of neurons in the latero-capsular division of the central nucleus of the amygdala, and this inhibition is impaired in an arthritis pain model [39] (Fig. 1).

### Connections of the PFC–Basal Nuclei

The cerebral cortex is connected to the basal ganglia/nuclei and thalamus by several cortico-basal nuclei-thalamo-cortical loops [17]. A longitudinal brain imaging study of patients with chronic back pain shows that increased functional connectivity of the PFC to the nucleus accumbens (part of the striatum, and input part of the limbic loop through the basal ganglia) is predictive of pain persistence [40]. In addition, the strength of synchrony or functional connectivity between the mPFC and nucleus accumbens is predictive of individuals who subsequently transit to chronicity 1 year later [41]. Furthermore, it was shown that greater functional connections between the dorsal mPFC-amygdala-accumbens circuit contribute to risk of chronic pain in subacute back pain patients [42].

The functional significance of the PFC–basal nuclei connection in chronic pain is supported by experimental animal studies. For example, a longitudinal awake rat fMRI study indicates that the PFC and nucleus accumbens display abnormal activity to normally innocuous stimuli at 28 days after peripheral nerve injury, coincident with the development of tactile allodynia [43] (Fig. 1).

## Neurochemistry of the PFC Related to Pain

### Interactions of Neurotransmitters in the PFC that Mediate Antinociceptive Effects

A series of studies have been conducted on the ventrolateral orbital cortex (VLO) of rats, that could yield insights into the interplay between different ‘classical’ neurotransmitters in the PFC that are important for antinociception. A projection from the VLO to the PAG is involved in antinociception in rats, and many studies indicate that the projection neuron could be inhibited by a GABAergic neuron, which in turn is inhibited by opioids or dopamine. Unilateral microinjection of glutamate into the VLO depresses the nociceptive, tail flick reflex. Bilateral microinjections of GABA into the ventrolateral region of the PAG eliminates this ventrolateral PFC-evoked inhibition of the tail flick reflex, indicating that the VLO plays an important role in modulation of nociception, and that this role is mediated by the PAG [44]. In addition, microinjection of morphine into the VLO produces antinociception, and this effect is attenuated by administration of the GABA agonist muscimol. Results suggest that morphine could act on opioid receptors to produce inhibition of GABAergic inhibitory interneurons in the PFC, leading to a disinhibitory effect on the VLO output to the PAG [45].

As with opioid receptors, dopamine D2-like receptor activation could attenuate the inhibitory action of GABAergic interneurons on neurons projecting to the PAG [46, 47]. Microinjection of a non-selective dopamine receptor agonist, apomorphine, or the dopamine D2 receptor (D2R) agonist, quinpirole, into the VLO attenuates mechanical allodynia in SNI rats. This effect is blocked by a D2R antagonist but enhanced by a D1R antagonist. Results suggest that the dopaminergic system is involved in mediating ventrolateral PFC-induced antinociception via activation of D2R in the PFC [48]. Endogenous release of dopamine in the rat PFC, induced by high-frequency stimulation of dopaminergic neurons in the ventral tegmental area (VTA), was found to significantly suppress nociceptive responses. Local microinjection of a selective D2R agonist to the PFC induces long-lasting suppression of mechanical nociceptive responses, while a D2R but not D1R antagonist impairs suppression [49].

Microinjection of a selective adrenoceptor agonist, methoxamine, into the VLO increases mechanical paw withdrawal threshold in a dose-dependent manner. This is antagonized by pre-microinjection of the selective α1 adrenoceptor antagonist benoxathian into the same site. Results suggest that activation of α1 adrenoceptors facilitates glutamate release and increases the activation of ventrolateral PFC output neurons that are projecting to the PAG, leading to descending antinociception [50].

### Glutamate

Increased neuronal excitation due to ionotropic glutamate receptor activation in the mPFC has generally been shown to result in antinociception. Administration of ionotropic glutamate agonists to the PFC has the effect of reducing pain in SNI rats, and infusions of d-cycloserine, a partial agonist of the NMDA receptor directly into the mPFC induces antinociception in these animals [51]. In addition, administration of drinking water containing d-Asp, a precursor of endogenous NMDA and a putative agonist at NMDA receptors, reduces mechanical allodynia, improves cognition and motor coordination, and increases social interaction in mice with neuropathic pain [52]. Direct injection of AMPAkines, which enhance glutamate signaling via α-amino-3-hydroxy-5-methyl-4-isoxazolepropionic acid (AMPA) receptors into the PFC, results in analgesia and potentiates morphine analgesia [53]. Unilateral microinjection of glutamate into the ventrolateral PFC of rats depresses the nociceptive tail flick reflex, whereas bilateral microinjections of GABA into the ventrolateral regions of the PAG eliminates this effect [54]. Likewise, local anesthesia of the infralimbic cortex or the adjacent prelimbic cortex increases nociception, suggesting that these areas are tonically active in anti-nociception [55].

Neuropathic pain induced by experimental SNI results in enhancement of glutamate release and increased synaptic vesicle proteins in the mPFC, which may be due to activation of ERK1/2- and CaMKII-synapsin signaling cascades in presynaptic axonal terminals [56]. The calmodulin-stimulated adenylyl cyclases, AC1 and/or AC8, are also important in mediating long-lasting enhanced presynaptic transmitter release in the ACC in mice with inflammatory pain [57]. The intrinsic excitability of excitatory neurons in the prelimbic cortex is decreased in rats with chronic pain, and knocking down cyclin-dependent kinase 5 reverses this deactivation, and has an antinociceptive effect. In addition, optogenetic activation of prelimbic excitatory neurons is also found to exert analgesic and anxiolytic effects in mice subjected to chronic pain, whereas inhibition is anxiogenic in naive mice [58].

In contrast to ionotropic glutamate receptors, a pro-nociceptive effect has generally been observed after activation of metabotropic glutamate receptors in the mPFC. Upregulation of mGluR5 is found in the prelimbic region of the mPFC of chronic neuropathic pain animals, and pharmacological blockade of mGluR5 in the prelimbic cortex ameliorates the aversive behaviors including tactile hypersensitivity and depressive-like activity [59]. In contrast, stimulation of mGluR5 in the infralimbic cortex enhances nociception in healthy controls and monoarthritic animals [55]. mPFC pyramidal neurons in SNI rats exhibit increased input resistance and excitability as well as mGluR5-mediated persistent firing [60], and both the prototypical mGluR5 agonist CHPG and a positive allosteric modulator for mGluR5 (VU0360172) increase synaptically evoked spiking in mPFC pyramidal cells [61]. mGluR5 agonists have also been reported to drive endocannabinoid signaling in the mPFC [62]. Group II mGluRs (mGluR2 and mGluR3) act on glutamatergic synapses to inhibit direct excitatory transmission onto pyramidal cells, resulting in reduced pyramidal cell output [63].

### GABA

Increased input to GABAergic neurons in the mPFC from other parts of the brain could lead to inhibition of principal neurons in the mPFC that project to the PAG and increased pain. The paraventricular nucleus of the thalamus sends a projection to the mPFC, which terminates on GABAergic inhibitory neurons. Inhibition of the paraventricular neurons attenuates visceral pain and induces activation of the descending pain modulation pathway. In contrast, activation of glutamatergic principal neurons in the mPFC reverses visceral nociception [30]. Inflammatory pain due to intra-plantar carrageenan injection increases GABA, resulting in cell deactivation of the pre-limbic cortex, whereas the local application of a GABA_A_ receptor selective antagonist, bicuculline, reduces mechanical allodynia [64]. Neuropathic pain resulting from peripheral nerve injury inhibits pyramidal cell firing in the prelimbic area of the prefrontal cortex as a result of feed-forward inhibition, mediated by parvalbumin-expressing GABAergic interneurons. In accordance, optogenetic activation of inhibitory archaerhodopsin or excitatory channelrhodopsin-2 in the GABAergic inhibitory neurons decreases and increases pain responses, respectively [65].

### Opioids

Activation of opioid receptors on GABAergic neurons could lead to disinhibition of neurons in the mPFC that are projecting to the PAG, and anti-nociception. A human positron emission tomography (PET) study demonstrates that opioid receptors are enriched in cortical projections of the medial pain system in the cingulate cortex and PFC. This suggests that the medial pain system is an important target for the antinociceptive effect of opioids [66]. In accordance, patients with central post stroke pain have reduced opioid receptor binding in the contralateral thalamus, parietal, secondary somatosensory, insular and lateral prefrontal cortices, ACC, posterior cingulate, and midbrain gray matter [67]. Patients with peripheral neuropathic pain show decreased bilateral and symmetrical opioid receptor binding, while those with chronic post-stroke pain show decreased opioid binding in the hemisphere contralateral to pain [68]. Rheumatoid arthritis, trigeminal neuralgia, central post-stroke pain, and acute experimental pain all lead to decreased binding of opioid ligands in pain processing regions such as the ACC, PFC, parietal and temporal cortex, amygdala, thalamus, caudate, pons, and cerebellum during the painful period, in contrast to pain free intervals or healthy subjects [69, 70]. The decreased opioid binding could indicate increased endogenous release of opioids, or possibly, receptor internalization or loss of neurons carrying these receptors [70]. μ-Opioid receptor selective radiotracer-labeled PET studies show that placebo effects are accompanied by increased opioid neural transmission in pain-sensitive brain regions, including the rostral ACC, PFC, insula, thalamus, amygdala, nucleus accumbens, and the PAG [71]. Stimulation of the primary motor cortex (M1) for the control of pain also induces changes in the endogenous opioid system, including changes in exogenous opioid ligand [^11^C] diprenorphine binding in the middle cingulate gyrus and PAG that are significantly correlated with pain relief [72].

In animals, unilateral microinjection of morphine into the ventrolateral PFC dose-dependently suppresses the tail flick reflex, and this effect is reversed by the opioid receptor antagonist naloxone [44]. In another study, the VLO is found to be involved in opioid-induced inhibition of neuropathic pain, with the effect mediated by μ-opioid receptors [73]. There is evidence that mPFC κ-opioid signaling can regulate the flow of emotional state information from the amygdala to the mPFC during pain-induced stress and anxiety [74]. A marked increase in κ-opioid receptor and prodynorphin mRNA expression is found in the PFC 14  days after chronic constriction injury of the sciatic nerve in mice [75]. Sleep impairments are a predictor of the occurrence of chronic pain, possibly as a result of changes in dopamine and opioid systems that play a role in endogenous pain inhibition [76].

### Cannabinoids

The cannabinoid signaling system plays a complex and somewhat controversial role in the regulation of pain. In animals, chronic constriction injury produces a significant decrease in cannabinoid receptor agonist WIN 55,212-2-stimulated [(35) S] GTPγS binding in membranes prepared from the ACC, suggesting desensitization of the cannabinoid 1 receptor (CB1) in the ACC in neuropathic pain [77]. An increase in endocannabinoid 2-arachidonoylglycerol level is found in the PFC and plasma of mice with experimental osteoarthritis, and peripheral blood lymphocytes of patients with osteoarthritis, compared with healthy subjects [78]. The excitation of mPFC neurons is inversely related to the activity of the amygdala during pain, and CB1 activation reduces the excitation of inhibitory neurons that are terminating on mPFC neurons, as a result of abnormally increased amygdala output [79]. Consistent with this model, the endocannabinoid, palmitoylethanolamide, reduces pain-related behaviors and restores glutamatergic synapses in the mPFC of neuropathic mice [80]. Furthermore, daily treatment with *N*-arachidonoyl-serotonin, a hybrid inhibitor of the endocannabinoid-catabolyzing enzyme, fatty acid amide hydrolase, and antagonist of vanilloid type 1 channel, restores cortical neuronal activity and reduces allodynia after sciatic nerve injury in rats [81].

### Dopamine

The PFC receives dopaminergic innervation from the VTA in the midbrain [17]. Close appositions are found between dopamine fibers and GABAergic interneurons in the ACC [82]. In addition, the dopamine D2/D3 receptor availability in the ventrolateral PFC has been reported to correlate with recalled efficacy of placebo, suggesting a role of dopamine in pain processing [83]. In animals, high-frequency stimulation of the VTA produces long-lasting suppression of nociceptive responses in the rat PFC, including the cingulate and prelimbic areas. A D2R but not a D1R antagonist impairs the long-lasting suppression evoked by high-frequency stimulation, suggesting that dopamine may modify PFC nociceptive responses via the D2R [49].

### Noradrenaline

The PFC receives noradrenergic innervation from the locus coeruleus in the midbrain [17]. This innervation is significantly increased in nerve-injured animals compared to controls [60]. Norepinephrine induces persistent firing of pyramidal neurons of the PFC. This effect is independent of recurrent fast synaptic excitation, but involves presynaptic α1-adrenoceptors that facilitate glutamate release [84]. The pain-inhibitory actions of antidepressant drugs may involve elevated noradrenaline concentrations in the dorsal horn, which occurs concurrently with activation of supraspinal facilitating systems that are dependent on α1-adrenoceptors in the mPFC [85]. Neuropathic pain-associated depression and anxiety have been associated with noradrenergic dysfunction including increases in locus coeruleus bursting activity, increases in tyrosine hydroxylase and noradrenaline transporter expression, and enhanced expression and greater sensitivity of α2-adrenoceptors in the locus coeruleus [86].

There is, however, evidence that noradrenergic neurons in the locus coeruleus could also participate in the development and maintenance of allodynia and hyperalgesia after nerve injury [85]. The activation of noradrenergic locus coeruleus neurons that are projecting to the PFC has been reported to result in exacerbation of spontaneous pain [87]. Whisker pad mechanical hypersensitivity after trigeminal neuropathy was found to be alleviated by immunolesioning of noradrenergic neurons in the locus coeruleus, or microinjection of the α1 adrenergic antagonist, benoxathian, into mPFC [88], suggesting a pronociceptive effect of excessive α1-adrenergic stimulation in the mPFC.

α2 adrenoceptors modulate the voltage-dependent activation of hyperpolarization-activated cyclic nucleotide gated (HCN) channels [89]. Systemic administration of a selective α2B receptor agonist, A-1262543, results in attenuation of mechanical allodynia in animals with spinal nerve ligation injury [90].

### Cyclic Nucleotide Gated Channels

These channels are regulated by α2-adrenergic receptors. Electrophysiological and behavioral studies demonstrate that HCN channels and the cAMP/protein kinase A signaling axis promote hyper-excitability and persistent firing in pyramidal neurons of the mPFC, in rats with neuropathic pain [60].

### Acetylcholine

Cholinergic neurons appear to play a role in mPFC function and pain. One week after nerve injury, cholinergic modulation of layer V pyramidal neurons is severely impaired. The cause of this impairment is a  60% reduction of an m1 cholinergic receptor-coupled, pirenzepine-sensitive depolarizing current [91]. Reciprocal interactions between cholinergic and opioidergic modulation likely impact the function and efficacy of both opioids and cholinomimetic drugs [92].

### *N*-acetylaspatate

*N*-acetyl aspartate (NAA) is the second-most-concentrated molecule in the brain after glutamate. It is present in neurons of the adult brain, but its exact function remains to be determined. In vivo single-voxel proton magnetic resonance spectroscopy studies show reductions of NAA and glucose in the DLPFC of patients with chronic back pain [93]. Reduced levels of NAA in bilateral DLPFC and increased levels of myo-inositol in the left orbitofrontal cortex were also found in a patient with chronic pain due to complex regional pain syndrome type I [94].

### Brain-Derived Neurotrophic Factor

Brain-derived neurotrophic factor (BDNF) is an extracellular growth factor that influences neuronal activity, function, and survival, primarily by activating the receptor protein kinase TrkB. Both neuronal BDNF and prodynorphin mRNA levels are increased in the ACC and PFC of mice subjected to chronic constriction injury of the right sciatic nerve [95]. A role of BDNF in supraspinal pain regulation is suggested by the observation of a reduction in BDNF levels in the infralimbic cortex, but not in the prelimbic cortex, 3 days after Complete Freund's Adjuvant induction of inflammatory pain. In contrast, BDNF infusion into bilateral infralimbic cortices alleviates inflammatory pain and accelerates long-term recovery from pain, coincident with increased regional c-fos activity [96]. These results suggest an antinociceptive effect of increased mPFC activity that is induced by BDNF.

## Changes in the PFC During Acute Pain

### Human Studies

fMRI studies show that the discrimination of pain intensity, a non-spatial aspect of pain, activates a ventrally-directed pathway extending bilaterally from the insular cortex to the PFC. This activation is distinct from a dorsally-directed activation of the posterior parietal cortex and right DLPFC that occurs during spatial discrimination of pain [4]. Using the technique of standardized low-resolution brain electromagnetic tomography, significant activation is found in the somatosensory, ACC, operculo-insular, and DLPFC cortices during pain [97]. In an electroencephalogram (EEG) study of healthy human subjects, the subjective perception of tonic pain was shown to be selectively encoded by gamma oscillations in the medial prefrontal cortex [11]. These oscillations encode pain intensity rather than spatial discrimination. In addition, analysis of EEG data after right and left hand stimulation reveals that the encoding of pain in the mPFC is independent of the side of stimulation. Moreover, the interpretation of noxious stimulus intensity as pain is associated with a change from spatially specific alpha and beta oscillations in sensorimotor areas to a spatially independent representation of pain by gamma oscillations in the mPFC [98]. In a human experimental model for abdominal pain, LPS-treated healthy subjects showed visceral pain and significant and transient increases in inflammatory cytokines, along with impaired mood and decrease in visceral pain thresholds. Pain sensitization is accompanied by increased activity in several brain regions, including the DLPFC [99]. A similar study demonstrated that human subjects injected with LPS became more sensitive to pain compared to the placebo group, and increased pain sensitivity is accompanied by greater activity in the anterior insular cortex, but decreased activity in the ventrolateral PFC and the rostral ACC [100]. When pain is controllable, the DLPFC has been shown to downregulate pain-evoked activation in important pain-processing regions. In contrast, sensitization during uncontrollable pain is mediated by increased connectivity between the anterior insula and the mPFC [101].

The PFC receives afferents from the visual and auditory association cortices that may have effects on antinociception. Functional connectivity between the frontal and parietal cortices and the rostral ACC/mPFC is positively associated with the effect of visual cues on pain ratings [102]. Likewise, auditory inputs have been reported to modulate pain perception, and this is dependent on an intact connectivity between the angular gyrus to the right DLPFC [103]. Acute periodontal pain stimulus elicited by a periodontal probe induced an increase in brain activity, as detected by brain oxygen utilization using functional near-infrared imaging (FNIRS), in all areas of the PFC [104].

Inter-individual differences in amount of cortical gray matter is inversely related to the level of visceral pain. It is found that increased sensitivity to rectal pain stimuli correlates significantly with reduced grey matter volume in several regions of the brain, including the thalamus, insula, posterior cingulate cortex, ventrolateral and orbitofrontal PFC, amygdala, and basal ganglia [105]. The cerebellum has also been shown to have a role in pain processing/modulation, possibly due to its extensive connections with the PFC and brainstem regions involved in descending pain control [106]. Analysis of human brain chemistry using single-voxel proton magnetic resonance spectroscopy shows that in healthy individuals, combined glutamine/glutamate levels pooled across pain-related brain regions are positively correlated with pain intensity, whereas no appreciable relationship with GABA is found [107].

The PFC is also involved in empathy for pain. Bilateral anterior insula, rostral ACC, brainstem, and cerebellum are activated when subjects receive pain and also by a signal that a loved one is experiencing pain [108]. Another study shows that empathy involves increased activation of the anterior insula and ACC as well as the mPFC and temporal pole when sharing other’s social suffering [109]. Ventro-mPFC responses to somatosensory representations of others’ pain are present even in patients with congenital insensitivity to pain [110]. Interestingly, recipients of social and emotional support have increased activity in the left lateral/mPFC and some temporal regions [111], which may help to produce anti-nociception, and coping with pain.

### Animal Studies

#### Changes in Global Gene Expression, Epigenetic Effects, and Posttranslational Modifications

Global gene expression studies have detected significant neuroimmune interactions after peripherally induced pain. Microarray analyses of differentially expressed genes in the PFC at 3 days after inflammatory pain induced by facial carrageenan injection showed ‘immune system process’ as the dominant ontology term. Increased mRNA expression of immune-related genes, S100a8, S100a9, Lcn2, Il2rg, Fcgr1, Fcgr2b, C1qb, Ptprc, Ccl12, and Cd52, were verified by RT-PCR. The mild neuroinflammation that occurs in the PFC during peripheral inflammation may actually result in anti-nociception. Facial carrageenan-injected mice showed significantly reduced nociceptive responses after injection of C terminus of murine S100A9 protein in the lateral ventricles and PFC but not somatosensory cortex [112]. Epigenetic changes have also been found in the PFC after pain. miRNA microarray analysis shows significantly increased levels of miR-155 and miR-223 in the PFC of carrageenan-injected mice, and changes in two of the miRNA targets, c/ebp Beta and granulocyte colony-stimulating factor (GCSF) were verified by real-time RT-PCR. Increase in some of these markers may similarly lead to neuroinflamamtion of the PFC [113].

Relatively little is known about posttranslational modification of proteins such as phosphorylation or glycosylation in the PFC during pain. In one study, rats with unilateral infraorbital nerve chronic constriction injury show bilateral trigeminal dynamic mechanical allodynia, and bilateral upregulation of pERK-1/2 in the ventro-mPFC 2 weeks after nerve injury, suggesting that these changes may contribute to altered pain perceptions [114]. Another study, however, found that inflammatory pain induces persistent p38 mitogen-activated protein kinase (P38) but not ERK or c-Jun N-terminal kinase hyperphosphorylation in the prelimbic cortex [115].

#### Changes in Glial Cells and Neuroinflammation

Glial cells respond or react to glutamate released from neurons, and in turn produce inflammatory mediators which could further increase neuronal excitation [116]. This may serve an adaptive function, in the sense that it could activate cortical areas such as the mPFC that are involved in supraspinal antinociception [112]. Over the long term, however, chronic neuroinflammation may lead to trimming of dendritic branches, decreased synaptic connections, reduction in pyramidal neuron output from the mPFC, and loss of antinociception. Peripheral nerve injury has been reported to activate microglia not only in the spinal cord but also the brain [117]. Neuropathic pain as a result of nerve injury is associated with increased IL-1β expression in the damaged nerve, dorsal root ganglia, spinal cord, brainstem, hippocampus, and PFC [118]. Expression levels of cytokines IL-10 and IL-1β are upregulated in the contralateral VLO of SNI rats [119], and IL-1β expression and glutamatergic neurotransmission are enhanced in the PFC of mice with neuropathic pain [120]. Memory deficits, depressive, and pain behaviors are modulated by peri-sciatic administration of IL-1β neutralizing antibody in rats or deletion of IL-1 receptor type 1 in mice. Increased expression of Toll-like receptor 4 (TLR4) is coupled with enhanced glial activation in the PFC and increased levels of proinflammatory cytokines. Moreover, central or peripheral administration of a TLR4-specific antagonist, TAK-242, attenuates visceral pain sensation [121]. TLR4 blockade counteracts the hyperalgesia phenotype present in mice fed on high-fat diet, indicating a role for this receptor in visceral pain [122].

It has been suggested that proinflammatory mediators (e.g., cytokines) and activation of microglia and astrocytes could be responsible for the structural, functional, and molecular neuroplasticity changes observed in supraspinal structures, associated with pathological pain [123]. Bilateral increases in the density of microglia are observed in the infralimbic cortex of rats 7 days post-injury, without any detectable change in the other investigated regions, namely the ACC, prelimbic, and agranular insular cortices [124]. Microglia are activated by both SNI and intravenous injection of recombinant rat IL-1β, and this effect is reversed by peri-sciatic administration of anti-IL-1β [125]. Expression of proinflammatory/M1 microglia is increased, and anti-inflammatory/M2 microglia is decreased, in supraspinal regions after inflammatory pain. A suppressor of microglial activation, minocycline, reduces M1 activation and increases M2 activation, and attenuates neuropathic pain [126]. Minocycline promotes M2 microglia polarization as evidenced by upregulation of CD206 and Arg1 [127]. Besides microglia, astrocytes are also activated during pain. A significant increase in glial fibrillary acidic protein (GFAP), a marker of astrocytes, as well as changes in level of glial glutamate or GABA transporters, and vesicular glutamate or GABA transporters are found in the amygdala during pain, which may alter the state of excitation or inhibition in this nucleus [128], leading to downstream effects on its cortical targets.

There is evidence that inflammatory mediators could cause an increase in neurotransmitter release and neuronal excitation. Increased levels of TNF-alpha protein level are found in the mouse ACC after inflammatory pain induced by hind paw injection of Complete Freund’s Adjuvant, and in vitro whole-cell patch-clamp recordings reveal that TNF-alpha significantly enhances synaptic transmission through increased probability of neurotransmitter release in the ACC [129]. Similarly, increased expression of IL-8 is found in the ACC during the chronic phases of complete Freud’s Adjuvant-induced peripheral inflammation, and in vitro whole-cell patch-clamp recordings show that IL-8 enhances synaptic transmission through increased probability of neurotransmitter release in ACC slices [130]. These results suggest that pain-induced cytokine release and neuroinflammation may predispose to increased neuronal excitability in the PFC. Such an increase in excitability has been found, in layer V mPFC pyramidal cells after peripheral inflammation [131]. The carbon monoxide-releasing compound (tricarbonyldichlororuthenium(II)dimer) or an heme oxygenase 1 (HO-1) inductor (cobalt protoporphyrin IX) exert potent anti-allodynic and anti-hyperalgesic effect in sciatic nerve injured mice, likely due to their effects on modulating microglial activation [132].

#### Potential Links with Chronic Pain

Continually increased neuronal activity as a result of untreated pain could lead to neuroinflammation, and loss of neuronal processes in the mPFC, resulting in decreased modulation of pain. Another way in which persistent inflammation in the PFC could cause the progression to chronic pain is that persistent activation of the mPFC could result in increased mPFC–nucleus accumbens connectivity, which has been found to be associated with the chronification of pain (see below). Animals with SNI have increased expression of IL-1β not only in the injured sciatic nerve but also in CNS regions that are closely associated with pain, memory, and emotion, including the spinal dorsal horn, hippocampus, PFC, nucleus accumbens, and amygdala [125]. SNI also produces mechanical allodynia and depressive-like behaviors, and increases the expression of microglial markers (Iba1, CD11b) and M1, proinflammatory markers (CD68, iNOS, IL-1β, TNF-α, and 8-OH-dG) in the PFC[127]. Daily intrathecal minocycline treatment to reduce microglial activation starting from POD0 for 2 weeks alleviated mechanical allodynia before POD3, and attenuated anxiety on POD9 [133]. Alterations in microglia and astrocytes in cortical and limbic brain regions have been suggested to be a cause of pain-induced emotional and cognitive impairments [134]. Hence, targeting supraspinal pro-inflammatory mediators might be a novel approach for the treatment of neuropathic pain [135].

## Changes in the PFC During Chronic Pain

### Changes in Structure

Chronic pain is defined as pain lasting more than 1–3 months. Many studies have shown that there is loss of grey matter in the PFC, as a result of chronic pain. Patients with chronic back pain have regional grey matter decreases in the bilateral mPFC extending to the ACC and the right mPFC extending to the orbitofrontal cortex [136]. In another study, patients with chronic back pain show 5–11% loss of cortical gray matter volume in bilateral DLPFC, as well as the right thalamus, which is equivalent to the gray matter volume lost in 10–20 years of normal aging [137]. Voxel-based morphometry analysis reveals significant decreases in grey matter density in areas associated with pain processing and modulation, i.e., the DLPFC, the thalamus and the middle cingulate cortex in patients with chronic low back pain [138]. Similarly, reduced gray matter volume is found in the right anterolateral PFC, the right temporal lobe, the left premotor cortex, the right caudate nucleus, and the right cerebellum gray and white matter, in patients with chronic low back pain due to prolapsed intervertebral discs [139]. A weak negative correlation exists between back pain intensity and grey matter volume in the left DLPFC, ventrolateral PFC, and ACC [140]. Furthermore, patients with ankylosing spondylitis back pain exhibit cortical thinning in the primary somatosensory, insular, ACC, anterior mid-cingulate cortices, and the supplemental motor area, and increased gray matter volume in the thalamus and putamen, compared to controls [141].

Atrophy of the ventro-mPFC gray matter in combination with reduced white matter integrity and connectivity to the basal ganglia is found in patients with chronic complex regional pain syndrome [142]. These patients show a positive correlation between pain and the volume of the left posterior hippocampus and left amygdala, but a negative correlation with the volume of the bilateral DLPFC [143]. Patients with fibromyalgia have threefold greater age-associated decrease in gray matter volume and significantly less gray matter density in the insula, cingulate, and medial frontal cortices and parahippocampal gyri, than healthy controls [144]. Those with chronic myofascial pain exhibit changes in grey-matter density of the right DLPFC that correlate with pain thresholds, i.e., the more atrophy, the lower pain threshold [145]. Patients with pain disorder show gray matter loss in the prefrontal, cingulate, and insular cortex [146], and those with neuropathic pain after spinal cord injury show significant differences in pain-related regions as well as regions of the classic reward circuitry, that is, the nucleus accumbens and orbitofrontal, DLPFC, and posterior parietal cortices [147]. These patients also show decreases of both metabolism and gray matter volume in the left DLPFC, as well as hypo metabolism in the mPFC and gray matter volume loss in bilateral anterior insula and subgenual ACC [148]. Patients with central post stroke pain show decreases in grey matter volume in comparison to healthy controls, involving secondary somatosensory cortex, anterior as well as posterior insular cortex, ventrolateral PFC and orbitofrontal cortex, temporal cortex, and nucleus accumbens [149]. The presence of pain symptoms is the main predictor of both grey matter volume and NAA levels in the left DLPFC of patients with chronic fatigue syndrome, i.e., more pain is associated with reduced grey matter volume in these patients [150].

Patients with trigeminal neuralgia show gray matter volume reduction in the primary somatosensory and orbitofrontal cortices, as well as the secondary somatosensory cortex, thalamus, insula, ACC, cerebellum, and DLPFC. Grey matter volume decreases in the ACC, parahippocampus, and temporal lobe are correlated with increasing disease duration [151]. These patients also demonstrate gray matter volume reductions in the ACC and mid-cingulate cortex, insula, secondary somatosensory cortex, M1, premotor area, and several regions of the temporal lobe [152]. Patients with temporomandibular disorder have decreased gray matter volume in the left ACC, in the right posterior cingulate gyrus, the right anterior insular cortex, left inferior frontal gyrus, as well as the superior temporal gyrus bilaterally [153]. Those with burning mouth syndrome show decreased grey matter volume in the mPFC [154]. Patients with neuropathic pain from recurrent herpes simplex virus infections have decreased gray matter density in the PFC and ACC [155].

Reduction of cortical gray matter is also found in patients with visceral pain. Patients with functional dyspepsia have decreased cortical thickness in the DLPFC, ventrolateral PFC, mPFC, anterior/posterior cingulate cortex, insula, superior parietal cortex, supramarginal gyrus, and lingual gyrus [156], and those with chronic pancreatitis have reduced cortical thickness in brain areas involved in pain processing [157]. Patients with irritable bowel syndrome show generally decreased gray matter density in different parts of the brain, including mPFC and ventrolateral PFC, posterior parietal cortex, ventral striatum, and thalamus [158], or cortical thinning of the left cuneus, left rostral middle frontal cortex, left supramarginal cortex, right caudal anterior cingulate cortex, bilateral insula [159], posterior parietal cortex, and DLPFC [160]. A negative correlation is found between cortical thickness with the severity and duration of pain in these patients [159], although one study reported that thicker right lateral orbitofrontal cortex is strongly associated with a decrease in pain inhibition [161].

Proton spectroscopy imaging shows that chronic neuropathic pain is associated with reduced free and bound proton movement, indicators of subtle anatomical changes, in the mPFC, ACC, and mediodorsal nucleus of the thalamus [162]. In addition, microstructural abnormalities have been found in the right ACC and mPFC of patients with chronic myofascial pain, that are related to pain intensity and the course of the disease, which could account for the loss of cerebral grey matter [163].

In animals, it has been reported that basal dendrites of pyramidal neurons from SNI rats are longer and have more branches and spines, than their counterparts in sham-operated animals [164]. Another study, however, found that 1 week after SNI, pyramidal cells in layer V of the rat mPFC show reduced responses to excitatory glutamatergic inputs by about 50%, as well as decreased frequency of spontaneous excitatory synaptic currents. The apical dendrites are also shorter and less complex in these animals [165]. It is possible that shorter dendritic branches may contribute to reduced grey matter volumes in patients with chronic pain.

### Changes in Activity

Changes in activity have been found in the PFC during chronic pain. Patients with chronic back pain show aberrant blood oxygenation-level dependent (BOLD) high-frequency dynamics in the mPFC which is correlated with altered functional connectivity to pain signaling/modulating brain regions [40]. These patients show significantly reduced blood flow in the PFC, but increased blood flow in the cerebellum [166]. Fibromyalgia patients show increased low- and high-frequency oscillatory activity [167], and increases in theta, beta, and gamma power along with a slowing of the dominant alpha peak in the PFC [167]. Augmented frontal theta activity in FM patients correlates with measures of tenderness and mean tiredness scores [168]. Source localization analysis in patients with migraine reveals increased activity in the somatosensory cortex, but reduced activation in the anterior mPFC [169]. The activity in the DLPFC is inversely correlated with secondary hyperalgesia in healthy controls, but such correlation is absent in patients with fibromyalgia [170]. Patients with migraine without aura have significantly increased pain-evoked potential amplitudes and activity in pain matrix regions such as the primary somatosensory cortex, but reduced activity in the orbitofrontal cortex [171]. Likewise, studies on regional blood flow by PET show that patients with atypical facial pain have decreased blood flow in the PFC in response to painful thermal stimuli [172].

Experimental animal studies show neurons in the prelimbic region of the mPFC increase firing rates after noxious stimulation in rats. Chronic pain suppresses both basal spontaneous and pain-evoked firing rates, but enhancing basal PFC activity with low-frequency optogenetic stimulation scales up prefrontal outputs to inhibit pain [173]. Whole-cell recordings also reveal a significant reduction in excitability of layer V cortico-PAG projecting neurons contralateral to chronic constriction injury in mice [174].

### Changes in Connectivity

The connectivity of other cortical areas to the PFC and its subsequent projection to the PAG are important for its function in antinociception. Significant negative correlations between pain ratings and PAG-ventro mPFC/rostral ACC connectivity is found in patients with chronic lower back pain, after a pain-inducing maneuver [175]. These patients show decreased connectivity of the mPFC to posterior constituents of the default mode network but increased connectivity to the insular cortex in proportion to the intensity of pain [176]. Large decreases in hippocampal connectivity with the mPFC are also found in patients with back pain, compared to those who recovered [177]. Decreased connectivity between pain- and sensorimotor brain areas is found in patients with fibromyalgia, suggesting that weaker coupling between these areas might contribute to less effective control of pain [178]. Patients with irritable bowel syndrome have increased long- and short-range functional connectivity in primary sensorimotor cortices, but decreased long- and short-range functional connectivity in the anterior midcingulate cortex, inferior parietal lobules, and decreased long-range connectivity in the right anterior insula, and decreased short-range connectivity in bilateral PFC, and mPFC regions including the subgenual ACC [179]. Women with primary dysmenorrhea have hypoconnectivity of the DLPFC, the ventral mPFC and the PAG [180]. Similarly, female migraine without aura patients have decreased functional connectivity among pain-related brain regions [181]. The connectivity of the PFC with the PAG is also decreased in patients with migraine compared to healthy individuals [182]. A study of the relationship between white matter changes and pain intensity and pain affect in elderly people shows that the presence of white matter changes is related to increased pain affect, but not with pain intensity [183].

The role of connectivity of the insula to the mPFC in chronic pain still needs to be clarified, although most studies found that, as with other cortical areas, reduced connectivity of the insula to the mPFC is associated with increased pain. Hypoconnectivity between anterior insula and mPFC and negative relation with the visual analogue scale is found in women with primary dysmenorrhea during menstruation [184]. The strength of mPFC-to-insula connectivity migraine without aura patients is also negatively correlated with pain intensity during migraine attacks. Results suggest that greater subjective intensity of pain during a migraine attack is associated with proportionally weaker mPFC-insula connectivity [185]. Compared with controls, patients with painful knee osteoarthritis show reduced functional connectivity between the right anterior insula and other areas of the pain network [186]. In addition, reductions of pain ratings under the influence of acupuncture are significantly correlated with an increase of connectivity between the insula and the mid-cingulate cortex [187]. Some studies, however, report that increased connectivity of the insula with the PFC is associated with increased pain. Women with chronic pelvic pain have increased levels of combined glutamine-glutamate within the anterior insula and greater anterior insula connectivity to the mPFC [188], and patients with rheumatoid arthritis show an increase in brain connectivity between the insula and PFC [189].

The cause of the altered connectivity between brain regions in pain is unknown. Nevertheless, diffuse abnormalities in the microstructure of white matter tracts and lower fractional anisotropy or connectivity have been found in tracts adjacent to the ventrolateral PFC and tracts coursing through the thalamus in patients with temporomandibular disorder. The reductions in brain functional anisotropy are associated with increases in mean diffusivity and radial diffusivity, which are markers of inflammation and edema [190]. Analysis of white matter microstructural differences of neuronal and non-neuronal origins shows non-neuronal (e.g., neuroinflammation) white matter density differences in the inferior fronto-occipital fasciculus and the splenium of the corpus callosum of patients with chronic musculoskeletal pain [191]. These results suggest that, as with grey matter regions, neuroinflammation could affect white matter tracts, leading to reduced connectivity to the mPFC and PAG, and decreased anti-nociception. It is to be noted however, that excessive connectivity between cortical areas could result in pain rumination. Individual differences in pain rumination in chronic pain patients have been correlated with functional connectivity between the mPFC and the posterior cingulate cortex, the retrosplenial cortex, the medial thalamus, and the PAG [192].

### Changes in Neurochemistry and Glial Cells

A survey of neurochemical changes in patients with chronic low back pain by proton magnetic resonance spectroscopy shows that chronic low back pain patients have reductions of (1) NAA in the DLPFC, right M1, left somatosensory cortex, left anterior insula, and ACC; (2) glutamate in the ACC; (3) myo-inositol in the ACC and thalamus; (4) choline in the right SSC; and (5) glucose in the DLPFC, compared to controls [193]. The translocator protein (TSPO) is associated with symptom severity and cerebral pain processing in patients with fibromyalgia. This protein is upregulated during glial activation, and compared to mixed/low TSPO affinity binders, high TSPO affinity binders rated more severe pain and fibromyalgia symptoms. Results are consistent with a glial-related mechanism of chronic pain [194].

In animals, inflammatory pain induces persistent p38 hyperphosphorylation in the prelimbic cortex. Inhibiting p38 phosphorylation in the prelimbic cortex reverses the aggravated nociceptive responses to the formalin test, implying that persistent hyperphosphorylation of p38 underlies aggravated nociceptive responses in rats with chronic inflammatory pain [115]. Transcriptome-wide RNA sequencing performed 6 months after nerve injury reveals 1147 differentially regulated transcripts in the PFC in nerve-injured vs. control mice, including those related to the NMDA receptor, neurite outgrowth, gliosis, vesicular release, and neuronal excitability [195]. Decreased global methylation is found in the PFC and amygdala, but not the visual cortex or thalamus in mice, six months after peripheral nerve injury [196]. In addition, long-term changes in DNA methylation and upregulation of a gene involved with synaptic function, synaptotagmin II, is detected in the PFC of mice after chronic pain [197]. Genome-wide DNA methylation assessed at 9 months post SNI and sham rats reveals a large difference in the DNA methylation landscape in the brain and a remarkable overlap (72%) between differentially methylated probes in T cells and the PFC [198].

Early life exposure to pain might predispose to later pain. This could occur through long-term changes in brain opioid receptors in the PFC and PAG, and may involve the gut microbiota [199] and glial cells. Painful stimuli in the neonatal period produces pain behaviors immediately after injury that persist into adult life, and is accompanied by an increase in glial activation in cortical areas that process or interpret pain. These results suggest a role of glial cells in the PFC, in the chronification of pain [200].

### Changes During Chronification of Pain

The corticostriatal circuit exerts a modulatory function in the acute pain state. Optogenetic activation of this corticostriatal circuit in rats gave rise to bilateral relief from peripheral nociceptive inputs. Activation of this circuit also provided important control for the aversive response to transient noxious stimulations [201, 202]. However, this pathway could potentially also lead to the chronification of pain. A longitudinal brain imaging study of subacute back pain patients who were followed over the course of 1 year showed that when pain persisted, brain gray matter density decreased. Initially, greater functional connectivity of the PFC to the nucleus accumbens predicted pain persistence, implying the corticostriatal circuitry is causally involved in the transition from acute to chronic pain [40]. Patients with a single sub-acute back pain episode have white matter fractional anisotropy differences that accurately predicts pain persistence over the next year. Local fractional anisotropy is correlated with functional connectivity between the mPFC and the nucleus accumbens, consistent with the finding that this circuit is important for the chronification of pain [203]. The cortico-amygdala projection also appears to play a role in pain chronification. A 3-year longitudinal study of subacute back pain patients indicates that greater functional connections between the dorsal mPFC-mygdala-accumbens circuit contributes to risk of chronic pain [42]. In animals, a longitudinal fMRI study in awake rats shows that innocuous air-puff stimuli evokes a distributed sensory network of activations, including contralateral somatosensory cortices, thalamus, insula, and cingulate cortex. After peripheral nerve injury, however, the PFC and nucleus accumbens display abnormal activities to normally innocuous stimuli, when such stimuli induce tactile allodynia [43]. Compared to other brain regions, the nucleus accumbens plays a dominant role in the global network in a chronic pain state, and it has been suggested that hippocampal hyperexcitability may contribute to alterations in synaptic plasticity within the nucleus accumbens and pain chronification [204].

Pain is experienced by the majority of patients with Parkinson’s disease. These patients have a loss of dopamine-containing neurons in the substantia nigra pars compacta, and the dopaminergic deficit in this disease lowers multimodal pain thresholds that are amenable’s to correction following L-DOPA treatment [205]. Parkinson disease symptom severity is negatively correlated with ACC-DLPFC connectivity, and impaired functional coupling to pain modulatory regions, with disease progression [206]. A reduction in brain dopamine signaling due to compensatory downregulation of dopamine receptors could also occur as a result of addiction. In this context, smoking status at baseline is found to be predictive of persistence of back pain one year from symptom onset, and back pain persistence by smoking is correlated with synchrony of fMRI activity between the mPFC and the nucleus accumbens. The strength of this synchrony decreased in subacute back pain or chronic back pain patients who ceased smoking [207]. Such alterations may involve downregulation of striatal D2 receptors as a result of use of addictive drugs or substances [208], and could lead to increased corticostriatal input. The release of dopamine via the VTA-projection is a key part of the brain reward pathway [208], and pleasurable stimuli, including reward, inhibit pain. A study on healthy participants who underwent fMRI while playing a wheel of fortune game with simultaneous thermal pain stimuli and monetary wins or losses showed that winning decreased pain perception compared to losing [209]. In animals, there is evidence that the reward pathway is, itself, affected by pain. Rats with visceral pain show depressed intracranial-self stimulation and extracellular levels of nucleus accumbens dopamine [210]. It has been suggested that changes in the brain reward pathway could have an impact on the proclivity for depression and suicide, in patients with chronic pain [211].

The cerebral cortex is connected to the basal nuclei and thalamus by several cortico-basal nuclei-thalamo-cortical loops. In the case of the mPFC/ACC, this involves: mPFC/ACC -> nucleus accumbens -> ventral pallidum/substantia nigra pars retiulata -> mediodorsal nucleus of the thalamus -> mPFC/ACC and possibly, insula. The PFC has been defined as the projection area of the mediodorsal nucleus of the thalamus, but this is no longer true in both directions [212]. In macaques, the mediodorsal nucleus also sends axons to cortical areas other than the PFC, such as the insular cortex [213–215]. Moreover, it is known that these loops do not only end in the cortical area of origin, but also in an adjacent area, i.e., they could form ‘spirals’ in addition to loops [216]. Hence it is conceivable that corticostriatal inputs originating from the mPFC may not only terminate in the mPFC, but also the insula. Based on this possibility, we postulate that one function of dopamine D2 receptors in the nucleus accumbens is to counteract the effects of excessive glutamatergic input from the mPFC—that through the above loop/spiral could lead to excessive stimulation of the thalamus and insula, which is the cortical area most commonly associated with the sensation of pain [7].

In this scenario, the mPFC could serve dual, opposing roles in pain: (1) it mediates antinociceptive effects, due to its connections with other cortical areas and serving as the main source of cortical afferents to the PAG for modulation of pain, and (2) depending on chronic neuroinflammatory processes, and the level of dopamine receptor activation (or lack of) in the VTA-nucleus accumbens reward pathway, it could induce pain chronification via its corticostriatal projection and possibly, the mPFC-basal nuclei-thalamo-mPFC/insula loop/spiral. More research is needed in this area (Fig. 1).

## Role of the PFC in Management of Chronic Pain

Chronic pain is best managed using a multidisciplinary, biopsychosocial approach. The goal is not only the reduction of pain but also to enable patients and their loved ones to cope with their pain (Table 1).

**Table 1:** Effect of biopsychosocial pain management strategies on PFC activity

No.	Therapy	Effect on PFC activity	Reference(s)
1	rTMS	Increased	[217]
2	tDCS	Increased	[218–221]
3	Antidepressants	Increased	[222]
4	Acupuncture	Increased	[223, 224]
5	Transcutaneous electrical nerve stimulation	Increased	[225]
6	Cognitive behavioral therapy	Increased	[226]
7	Mindfulness	Increased	[227–229]
8	Music	Increased	[230]
9	Physical activity and exercise	Increased	[231]
10	Viewing partner pictures	Increased	[232]
11	Meditation	Decreased in thalamus and PFC	[233–235]
12	Religious prayer	Decreased in large parietofrontal network	[236]

### rTMS

Repetitive transcranial magnetic stimulation (rTMS) of the PFC or M1 is a non-invasive strategy that could have the potential to relieve chronic pain. rTMS stimulation of the left DLPFC in healthy subjects is found to exert a bilateral control on pain system. rTMS is also associated with increases in thermal and mechanical pain thresholds, compared to sham treatment [237]. Similarly, left DLPFC-rTMS delivered at 10 or 20 min after capsaicin application significantly decreases spontaneous pain in both hands, whereas no significant effect is reported after right DLPFC rTMS [238]. rTMS is associated with increased activity in the posterior cingulate gyrus, precuneus, right superior frontal gyrus, right insula, and bilateral postcentral gyrus. Activity in the right superior PFC is negatively correlated with pain ratings [217]. rTMS-induced analgesia is associated with elevated blood oxygenation-level dependent (BOLD) signal in Brodmann areas 9 and 10, and diminished BOLD signal in the ACC, thalamus, midbrain, and medulla [239]. Patients with fibromyalgia who received active TMS over 2 weeks showed a 29% reduction in pain as well as improvement in depression symptoms, compared to baseline [240]. Those with chronic migraine experienced a significant reduction of the frequency of attacks during treatment by high-frequency rTMS over the left DLPFC [241], and a short-course of rTMS over the left DLPFC was found to alleviate mild traumatic brain injury-related headache symptoms [242]. Other studies, however, indicate that stimulation of M1 is more effective than the DLPFC in producing an antinociceptive effect in patients with migraine [243]. Patients with burning mouth syndrome who received rTMS showed decreases in pain intensity by 67% compared to baseline [244]. rTMS may also be useful for the management of visceral pain. Patients with gastric bypass surgery who received rTMS over the left PFC showed 36% less patient-controlled morphine pump use compared to sham-treated controls [245].

The antinociceptive effect of rTMS likely involves activation of opioid receptors. A single session of postoperative prefrontal rTMS is associated with a reduction in patient-delivered morphine use after gastric bypass surgery [246]. In addition, rTMS of area 9 of the left DLPFC produces analgesic effects on postoperative and laboratory-induced pain, but this effect was reduced by pretreatment with an opioid receptor antagonist, naloxone, suggesting that rTMS-induced analgesia requires opioid activity [247]. Moreover, rTMS reduces μ-opioid receptor availability compared to sham rTMS suggesting release of endogenous opioids in the right ventral striatum, medial orbitofrontal, PFC, ACC, left insula, superior temporal gyrus, DLPFC, and precentral gyrus [248]. In another study, naloxone injection significantly decreased the analgesic effects of M1 stimulation, but did not change the effects of rTMS to the DLPFC, or sham rTMS. This suggests that the analgesic effects induced by the rTMS at these two cortical sites are mediated by different mechanisms [249].

The analgesic effects of M1 or DLPFC rTMS are also decreased by ketamine. Since ketamine is an inhibitor of NMDA receptors, this suggests that rTMS-induced antinociception is dependent on NMDA receptors and may involve long-term potentiation (LTP) mechanisms [250]. Lipidomic analysis of the PFC of rats after rTMS indicate loss of plasmalogen species with long-chain polyunsaturated fatty acids, with an increase in their corresponding lysophospholipids, suggesting endogenous release of long-chain fatty acids such as docosahexaenoic acid (DHA) after rTMS [251]. DHA may be metabolized to resolvin D1 by 15-lipoxygenase-1 (Alox15), which has been shown to play an important role in LTP [252, 253] and antinociception [252, 253] . Results suggest that LTP-like mechanisms likely play a role in the antinociceptive effects of rTMS [254], possibly through the involvement of endogenous opioids and fatty acids (Table 1).

### Transcranial Direct Current Stimulation

Anodal transcranial direct current stimulation (tDCS) to the DLPFC has been shown to improve symptoms in a range of domains including working memory, mood, and pain perception [218–220]. tDCS of the left DLPFC in healthy subjects induces increased perfusion in brain regions that are anatomically connected to the DLPFC [221]. The beta band power at EEG is increased, and the alpha band power is decreased after anodal tDCS [255]. tDCS to the left DLPFC induces an analgesic effect, which is correlated with reduced perfusion to the posterior insula and thalamus [256]. tDCS is associated with a 23% reduction in patient-controlled opioid usage after lumbar surgery, and a 31% reduction is observed in pain-at-its-least ratings from admission to discharge [257]. Likewise, patients who received real tDCS report no pain exacerbation or worse mood after total knee arthroplasty, compared with those who received sham treatment [258]. Patients with chronic pain due to myofascial pain syndrome report decreased mean visual analog scale values, after tDCS to the DLPFC [259]. Anodal tDCS over the left DLPFC ameliorates neuropathic pain in patients with multiple sclerosis [260], and tDCS stimulation of either left M1 or DLPFC reduces headache and pain intensity in patients with migraine [261]. Other studies, however, found that tDCS over M1 but not DLPFC produces pain relief in patients with diabetic neuropathy [262]. Ratings for others’ pain also increased in subjects that received anodal tDCS of the DLPFC, consistent with a role of the PFC in empathy for pain [263]. There is evidence that analgesic effects of M1-tDCS may involve endogenous μ-opioids [264] (Table 1).

### Antidepressants

A variety of medications are used to treat chronic pain, including gabapentin, pregabalin, noradrenaline, or serotonin-noradrenaline reuptake inhibitor antidepressants including duloxetine, tricyclic antidepressants such as amitriptyline, capsaicin, lidocaine patches, tramadol, botulinum toxin, and opioids. Rectal pain induces significant activation of the perigenual ACC, right insula, and right prefrontal cortex. Treatment with the tricyclic antidepressant, amitriptyline, is associated with reduced pain-related cerebral activations in the perigenual ACC and the left posterior parietal cortex, but only during stress [265]. In another study, duloxetine treatment is associated with a significant reduction in brain responses to painful stimulation in major depressive disorder patients in regions generally showing abnormally enhanced activation at baseline. Clinical improvement is associated with pain-related activation reductions in the pregenual anterior cingulate cortex, right prefrontal cortex, and pons, but interestingly, increased baseline activations in the right prefrontal cortex and reduced deactivations in the subgenual anterior cingulate cortex predicted treatment responders at week 8 [222]. Changes related to Regulator of G protein signaling 4, a signal transduction protein that controls the function of monoamine, opiate, muscarinic, and other G protein-coupled receptors, have been detected in the brain, after antidepressant treatment [266]. In another study, reductions in clinical pain scores during treatment with milnacipran, a selective serotonin and norepinephrine reuptake inhibitor (SNRI), in fibromyalgia patients are correlated with decreased baseline functional connectivity between the insula and ACC [267].

In animals, chronic treatment with tricyclic antidepressant, amitriptyline, results in altered somatic nociceptive responding following peripheral nerve-injury, and these effects are associated with a reduction of glial activation and suppression of neuroinflammation [268]. Treatment of rats with tapentadol, a norepinephrine reuptake inhibitor with μ-opioid receptor agonistic activity, has recently been introduced for the treatment of moderate to severe pain, which results in a dose-dependent increase in cortical dopamine and norepinephrine levels with a mean maximum increase of 600 and 300%, respectively [269]. Mice that have been treated with the antidepressant, tianeptine, show abrogation of downregulation of BDNF and p-CREB in the mPFC and hippocampus [270]. Treatment of mice with the noradrenaline-reuptake (NRI)/tricyclic antidepressant, maprotiline, results in increased expression of an enzyme, calcium independent phospholipase A_2_ (iPLA_2_), that is related to its antidepressant-like [271] and antinociceptive effects [272]. iPLA2 acts on membrane phospholipids to release DHA, and the latter is metabolized by Alox15 to resolvin D1. Both DHA and resolvin D1 have anti-inflammatory properties, and Alox15 plays a role in hippocampo-PFC LTP, and has antinociceptive effects in a mouse partial infraorbital nerve ligation model of neuropathic orofacial pain [252, 253] (Table 1).

### Acupuncture

Acupuncture has long been used for the control of pain. Patients with painful osteoarthritis who were scanned by PET show that real acupuncture and placebo (with the same expectation of effect as real acupuncture) results in greater activation of the right DLPFC, ACC, and midbrain, compared to skin prick (with no expectation of a therapeutic effect) [223]. fMRI analysis shows that acupuncture induces activation of the amygdala and ACC, PAG, and hypothalamus and relatively persistent activation of the anterior insula and PFC [224]. Patients with chronic back pain had reduced connectivity within the pain network, including the DLPFC, mPFC, ACC, and precuneus, but after acupuncture, connectivity was restored almost to the levels of healthy controls, and was correlated with a reduction in pain [273]. In patients with carpal tunnel syndrome, electroacupucture at distal acupoints results in PFC activation and reduction in pain [274]. The analgesic effects of acupuncture may involve the release of endogenous opioids in the brain [275] (Table 1).

### Transcutaneous Electrical Nerve Stimulation

Patients receiving low-frequency transcutaneous electrical nerve stimulation (TENS) show significant decreases in perceived pain intensity, which are correlated with pain-specific activation of the contralateral thalamus, PFC, and the ipsilateral posterior parietal cortex [225]. Furthermore, connectivity between the lateral PFC and the PAG is increased by TENS, which could result in antinociception [276].

### Cognitive Behavioral Therapy

A variety of psychological conditions can predispose to pain, including depression, anxiety, post-traumatic stress syndrome, somatoform disorders, addiction, and problems with personality. Some of these conditions may benefit from psychotherapy, including cognitive behavioral therapy, mindfulness training, acceptance and commitment therapy, making meaning, and counselling. Psychological factors (Axis 2 factors) commonly co-occur with physical causes (Axis 1 factors) in patients presenting with chronic pain. Cognitive behavioral therapy results in increased activation of the ventrolateral PFC and lateral orbitofrontal cortex, suggesting that therapy changes the brain’s processing of pain [226]. In another study, patients with mixed chronic pain types have decreased pain catastrophizing after 11 weeks of cognitive-behavioral therapy, that is associated with increased grey matter volume in the left DLPFC and ventrolateral PFC, right posterior parietal cortex, somatosensory cortex, and ACC [277].

### Mindfulness

Mindfulness practitioners but not controls are able to reduce pain unpleasantness and anticipatory anxiety during a mindful state. This is associated with increased rostral ACC activation during the anticipation of pain [227]. In other studies, reduction in pain during mindfulness is associated with activation of brain regions including the orbitofrontal, subgenual ACC, anterior insular cortex, and the DLPFC [228, 229].

### Music

Music modulates pain responses in different parts of the CNS. Brain regions with increased activity when listening to pleasurable music include the DLPFC, PAG, and rostral ventromedial medulla, which are parts of the descending pain modulatory system [230]. In another study, a significant increase in the amplitude of low-frequency fluctuations of the BOLD signal is detected in the left angular gyrus after listening to music, which correlates with increased functional connectivity with the right DLPFC and analgesia [103].

### Physical Activity and Exercise

Greater physical activity is associated with decreased pain ratings to repeated heat stimuli in patients with fibromyalgia [278]. These patients also show decrease in pain sensitivity and significantly greater activity in the anterior insula and the left DLPFC after exercise [231].

### Viewing Partner Pictures

Viewing pictures of a romantic partner reduces experimental pain, which is suggested to involve the brain reward pathway [279]. This is associated with reduced pain-related neural activity in the dorsal ACC and anterior insula, but increased activity in the ventro-mPFC and reduced self-reported ratings of pain [232].

### Meditation

In contrast to mindfulness, meditation appears to have an effect on decreasing activity in different parts of the brain. Meditators show lower pain-evoked event-related potentials in the midcingulate cortex, and lower unpleasantness ratings compared to controls, and this response is predicted by lifetime meditation experience [233]. Pain patients who have learnt Transcendental Meditation and practiced it for 5 months show decreased activity in the brain, including a 40–50% reduction in the thalamus and the PFC [234]. Likewise, practitioners of Zen meditation have reduced activity in executive, evaluative, and emotion areas, including the PFC, amygdala, and hippocampus during pain, compared with controls [235] (Table 1).

### Religious Prayer

In contrast to the most of the above mechanisms, but perhaps similar to meditation, religious prayer reduces the activity of a large parietofrontal network to produce an antinoiceptive effect. Naloxone has no significant effect on pain ratings or neural activity, suggesting that the effect is not opioid related. These results suggest that prayer may attenuate pain through a reduction in processing of pain stimulus saliency, rather than through known descending pain inhibitory systems [236] (Table 1).

## Changes in the PFC During Placebo Analgesia

Placebo was originally conceptualized as a commonplace method or medicine prescribed in order to please the patient and not because of its efficacy. In recent years, however, the placebo effect has been a topic of considerable interest, and the psychosocially induced changes in the patient’s brain and body may, in turn, affect the course of the disease [280]. Placebo analgesia is associated with decreased activation of first-order pain sensory areas, but increased activation of the DLPFC and mPFC. fMRI experiments show that placebo analgesia is related to decreased brain activity in pain-responsive brain regions, such as the thalamus and insula, but increased activity in the PFC [281]. Placebo response can be identified a priori in chronic back pain, in that neuronal interactions between pain processing regions (bilateral insula) and prefrontal regions (left mPFC) predetermine the probability of placebo response in chronic pain patients [282]. A study on esophageal pain in healthy adults that received placebo reveals large reductions in activity of the thalamus, somatosensory cortices, insula, and ACC, but increased activity in the ventrolateral PFC, which correlates with decreased pain extent and visual analog scale values [283]. Moreover, low-frequency rTMS that transiently disrupted left and right DLPFC function completely blocked placebo analgesia in volunteers subjected to a heat-pain paradigm, highlighting the importance of the DLPFC in the placebo response [284].

Placebo effects and expectations for reduced pain elicit reliable increases in activation in the PFC and the PAG [285], and placebo analgesic effects are positively correlated with fractional anisotropy in the right DLPFC, left rostral anterior cingulate cortex, and the PAG [24]. In addition, greater left hemisphere DLPFC to PAG connectivity and right hemisphere, thalamus to DLPFC connectivity are predictive of future placebo responses [286]. Significantly greater couplings are found between brain regions involved in cognitive processes [287], and between these areas and the brainstem and spinal cord, during placebo analgesia [288]. Placebo analgesia may be limited to the location at which pain relief is expected, suggesting spatial specificity in the pain modulatory pathway [289]. Besides the mPFC—PAG connection, the PFC—ventral striatum connection is also important for the placebo response.

In aversive and appetitive reinforcement learning, learned effects show extinction when reinforcement is discontinued. However, during placebo analgesia, little extinction occurs, and effects persist after the discontinuation of reinforcement. This has been found to be related to the suppression of prediction error processing in the ventral striatum by the prefrontal cortex [290]. There is evidence that the efficacy of placebo response might be predicted based on baseline PFC structure and function. In patients with dementia, the placebo analgesic response can be significantly diminished, when neurodegenerative changes are prominent in the PFC [280]. Moreover, reduction in migraine days after placebo treatment was significantly associated with the baseline gray matter volume of the mPFC which could predict post-treatment groups with high accuracy [291].

PET studies using the μ-opioid selective radiotracer, [^11^C]carfentanil, show that the placebo effect is associated with activation of endogenous opioid neurotransmission in different brain areas such as the ACC, orbitofrontal PFC and DLPFC, anterior and posterior insula, nucleus accumbens, amygdala, thalamus, hypothalamus, and PAG [292–294]. Placebo-induced activation of μ-opioid receptor-mediated neurotransmission is found in both higher-order and sub-cortical brain regions, which include the pregenual and subgenual rostral ACC, DLPFC, insular cortex, and the nucleus accumbens [295]. The use of a placebo results in reduced pain in 35% of patients, and almost the same percentage (36%) of patients respond to treatment with morphine. This suggests a close relation between the placebo effect and the opioid system [296]. In addition, the dopaminergic innervation of the PFC from the VTA could play a role in the placebo effect [297].

## Links Between Pain and Depression, Anxiety and Cognition, and Potential Reversibility of Chronic Pain-Induced Changes in the PFC

### Link with Depression

The presence of depressive mood may increase pain sensation and vice versa. Clinical studies and animal models have demonstrated that this interaction appears to involve the PFC. For example, the effect of mood on pain perception was demonstrated in an experimental pain study of female patients suffering from major depressive disorder. Compared to control subjects, depressed patients showed hyper activation in the left ventrolateral thalamus, the right ventrolateral PFC and DLPFC, as well as a stronger parametric BOLD signal increase in the right ventrolateral PFC, DLPFC, and in the contralateral insula, after painful stimulation [298]. Symptoms of depression and the presence of major depressive disorder are also associated with the magnitude of pain-evoked neuronal activations in brain regions associated with affective pain processing, such as the amygdala and contralateral anterior insula [299]. In addition, healthy subjects who report the largest increase in pain unpleasantness after sad mood induction show greater activation of the inferior frontal gyrus and amygdala, indicating a correlation between emotional regulation and pain affect [300]. State depression scores of chronic pain patients are also correlated with changes in the thalamus and the cingulate, DLPFC, and hippocampal cortices [301]. In patients with joint pain, there are significant correlations between Beck Depression Inventory scores, mPFC activation, and signals in limbic areas, suggesting that these areas are important in mediating the link between pain and depression [302]. A correlation also exists between Beck Depression Inventory scores and pain in patients with burning mouth syndrome [154].

In animals, long-term neuropathic pain at 28 days after injury results in depressive and anxiogenic-like behaviors, that are even more intense than the averseness associated with the primary pain stimulus [86]. Furthermore, rats with a depression-like history display increased ongoing pain behavior in the formalin test, although their thermal pain thresholds are unchanged [303]. Rats that show increased depression-like behaviors after neuropathic injury display higher levels of proinflammatory cytokines (e.g., IL-1β, IL-6) as well as imbalance of pro/anti-inflammatory cytokines, compared with rats without depression-like phenotype and sham-operated rats [304]. In addition, a substantial number of signaling pathway-associated genes with similar changes in expression have been found by next-generation RNA sequencing and pathway analysis, in mice with SNI and those subjected to chronic unpredictable stress. This suggests common molecular pathways in the PFC between pain and chronic stress/depression [305, 306].

### Link with Anxiety

Pain is often associated with anxiety, but whether these two entities are present in the same brain structure is less well-known. Human studies suggest that analgesic and anxiolytic effects could be served by distinct neural mechanisms and that multiple pathways are operating in the top-down modulation of pain [307]. In contrast, animal studies have provided evidence for overlapping pain/anxiety pathways. For example, it has been suggested that activation of ERK1/2 in the mPFC may contribute to the process of stress-induced cognitive and emotional disorders, leading to an increase in pain sensitivity [308]. Another study has found that optogenetic activation of prelimbic excitatory neurons exerts analgesic and anxiolytic effects in mice subjected to chronic pain, whereas inhibition is anxiogenic in naive mice [58].

### Link with Cognition

Many structural and molecular imaging studies highlight the extent of damage the brain sustains when patients experience prolonged, unrelieved chronic pain [309]. An fMRI study demonstrates that cognitive demands decrease pain-related brain activation. Attenuation of pain-related activity occures in the primary and secondary somatosensory cortices and the anterior insula when patients are engaged in cognitive tasks [310]. Inversely, painful stimuli may affect cognition—the mPFC and the mediodorsal nucleus of the thalamus form interconnected neural circuits that are important for spatial cognition and memory, and in chronic pain states, the mPFC is deactivated and mPFC-dependent tasks such as attention and working memory are impaired [311]. Patients with chronic low back pain show significantly less activation in the cingulo-frontal-parietal cognitive/attention network including the right DLPFC, the ACC, and bilateral superior parietal cortex during an attention-demanding multi-source interference task, compared to healthy controls [312]. Those with fibromyalgia have pain scores that are negatively correlated with grey matter values in the mPFC, whereas performance in a non-verbal working memory task is positively correlated with grey matter values in the left DLPFC [313]. Patients with complex regional pain syndrome have reduced cortical thickness in the DLPFC and the ventro-mPFC compared to healthy controls. This correlates with executive dysfunction, perseveration errors on the Wisconsin Card Sorting Test, and longer stop-signal task reaction times [306]. The number of words generated during the verbal fluency task in patients with somatoform pain disorder are significantly fewer than controls, and is correlated with a reduction in activated area of the PFC [314]. Patients with mild traumatic brain injury and severe pain in the hour preceding testing have reduced mid-DLPFC activation during a working memory task and poorer performance on the task [315]. Pain reduces cognitive task consistency/speed in P-type individuals who are distracted by pain relative to A-type, non-distracted subjects, and this disparity is related to differences in functional connectivity involving the DLPFC [316]. There is evidence that orofacial pain affects cognition. Patients with temporomandibular disorder perform poorly in neuropsychological tests of cognitive function, and have sluggish Stroop reaction times for all Stroop tasks [317].

Considering this bidirectional relationship between pain and cognition, it has been suggested that the level of cognition could be used to predict responsiveness to pain treatment. Patients who were studied 6 and 12 months after surgery show that premorbid limited cognitive flexibility and memory capacities are associated with pain chronicity and probably also its neuropathic quality [318]. Conversely, an improvement in attention test is found to be a reliable predictor of the treatment response, even before there is a reduction in the perception of pain [319]. Not all cognitive tasks involving the PFC are affected by pain, however, and some pain patients are able to perform certain working memory tasks as well as healthy subjects [320]. Pain and cognitive functions may also not be present in identical regions of the mPFC. Pain has been reported to be localized ventral to the cingulate sulcus while cognition is more dorsally placed in the ACC [321]. In addition, collation of data from different studies indicates that pain representations are localized to the anterior midcingulate cortex, negative emotion representations to the ventromedial prefrontal cortex, and cognitive control representations to portions of the dorsal midcingulate cortex [322].

Animal models provide further evidence for the negative effect of pain on cognitive functions of the PFC. Inflammatory pain disrupts orbitofrontal neuronal activity and risk-assessment performance in a rodent decision-making task [323], and causes impairment of spatial working memory performance that is associated with changes in the activity of the mPFC-medial dorsal nucleus of the thalamus circuit [31]. In the SNI model of neuropathy, chronic pain reduces the overall amount of information flowing in the fronto-hippocampal circuit [324]. Chronic pain also impairs cognitive flexibility and engages novel learning strategies in rats [325]. Surgical incision-induced nociception reduces the level of synaptic NMDA receptor 2B in the mPFC of mice, and this reduction has been suggested to contribute to a loss of cognition [326]. In addition, mice with SNI-induced pain demonstrate an increase of the insoluble form of Aβ1-42 in the hippocampus and display cognitive impairments and depression-like effects. These changes were normalized by administration of d-aspartate [327].

### Potential Reversibility of Chronic Pain-Induced Changes in the PFC

There is evidence that chronic pain-associated loss of gray matter in the PFC and the associated decrease in cognitive function may be reversible after successful treatment of pain. Individuals from the general population with ongoing pain show regional decreases in gray matter volume in the cingulate, PFC, and motor/premotor cortices, whereas no gray matter volume decreases are found in the group with pain that has stopped for > 12 months [328]. In addition, another study shows that patients with chronic lower back pain have reduced cortical thickness in the left DLPFC, but that cortical thickness is restored after pain treatment [329]. Partial recovery of abnormal insula and DLPFC connectivity to cognitive networks is found in patients after treatment of chronic low back pain [330]. Compared with healthy controls, patients with chronic pain due to hip osteoarthritis show gray matter decreases in the ACC, right insular cortex and operculum, DLPFC, amygdala, and brainstem, but patients who were pain-free after total hip replacement surgery showed gray matter increases in the DLPFC, ACC, amygdala, and brainstem [331]. Likewise, compared to controls, patients with chronic pain due to unilateral coxarthrosis have significantly less gray matter in the ACC, insular cortex and operculum, DLPFC, and orbitofrontal cortex, but when the patients were pain-free after recovery from endoprosthetic surgery, gray matter increases were found in nearly the same areas [332].

Longitudinal analysis of cortical thickness in patients with osteoarthritis of the knee and treated with acupuncture indicates that those who received verum acupuncture had greater thickness of the left posterior mPFC and reduced pain, compared to controls who received sham acupuncture [333]. Reduced connectivity between the DLPFC, mPFC, ACC, and precuneus was found in patients with chronic low back pain than in healthy controls, but after acupuncture, reductions in clinical pain were correlated with increases in connectivity [273]. A similar result is found in children with complex regional pain syndrome who were treated with intensive interdisciplinary psychophysical pain treatment. This resultes in reduced pain and increased gray matter in the DLPFC, thalamus, basal ganglia, amygdala, and hippocampus, and enhanced functional connectivity between the DLPFC and the PAG [334]. Reduced hyperconnectivity from the amygdala to multiple cortical/subcortical regions is found in chronic pain patients after pain rehabilitation treatment [33]. Interestingly, cognitive behavioral therapy has also been found to result in increased gray matter in patient with chronic pain [277]. These results suggest that decreases in gray matter and alterations in functional connectivity in chronic pain do not reflect permanent brain damage, but rather, are a reversible consequence of chronic nociceptive transmission, which normalizes when the pain is successfully treated [331].

## A Perspective and Conclusion

Pain is the main (if not the unique) sign which induces a person to consult a physician. It is also extremely important from an adaptive point of view, leading to behaviors in which the PFC and basal ganglia circuits play a fundamental role in selecting the most appropriate action module. PFC activity is a consequence of a perceptual cortical activity (a cognitive activity) in which pain information (pain stimuli or nociceptive stimuli) are integrated with other information including memories, mood, spatial awareness, and many more. The perceptual result, or result of this integration processing is, in turn, utilized to control pain at the peripheral level through PAG efferent signals toward dorsal horn neurons (or brainstem neurons). At this level, PAG signals are utilized to modulate nociceptive stimuli, i.e., still not perceptual nociceptive stimuli. This loop shows how on one side a sensory stimulus is transformed into a pereptual signal through high brain processing activity, and how perceptual activity is utilized to control the flow of afferent sensory stimuli at their entrance (dorsal horn) into the CNS. Many studies indicate that chronic pain is associated with loss of gray matter in the PFC, but that these changes may be potentially reversible, upon successful treatment of pain. They point to the importance of prevention and treatment of chronic pain, and associated depression, anxiety, and loss of cognition.

The management of chronic pain is particularly amenable to holistic approaches that are embodied by the biopsychosocial model of management. In this context, it has been suggested that cognitive brain networks may be exploited for the treatment of chronic pain [335]. Bearing in mind that the best way to prevent chronic pain is to inhibit the persistence and stabilization of acute pain, early interventions should be performed to mitigate the identifiable causes of the acute pain. This would then prevent aberrant plasticity from occurring in the CNS. Future studies are also needed to identify the spectrum of effective interventions, which may include utilizing nutraceuticals, physical and mental exercise, intermittent fasting, or other strategies to strengthen PFC function. Together with social support, cognitive behavioral therapy, and other measures to improve the functioning of the brain reward pathway, these approaches have the potential to take advantage of our emerging knowledge of the role of functional connectivity in the regulation of pain experience. It is likely that this will then provide methods for prevention and management of chronic pain that are more effective than the current standard of medical care.

## References

[1] Ongur D, Price JL (2000). The organization of networks within the orbital and medial prefrontal cortex of rats, monkeys and humans. Cerebral cortex.

[2] Goldman-Rakic PS (1996). The prefrontal landscape: implications of functional architecture for understanding human mentation and the central executive. Phil Trans R Soc Lond B.

[3] Apkarian AV, Bushnell MC, Treede RD, Zubieta JK (2005). Human brain mechanisms of pain perception and regulation in health and disease. Eur J Pain.

[4] Oshiro Y, Quevedo AS, McHaffie JG, Kraft RA, Coghill RC (2009). Brain mechanisms supporting discrimination of sensory features of pain: a new model. J Neurosci.

[5] Bastuji H, Frot M, Perchet C, Magnin M, Garcia-Larrea L (2016). Pain networks from the inside: spatiotemporal analysis of brain responses leading from nociception to conscious perception. Hum Brain Mapp.

[6] Wiech K, Jbabdi S, Lin CS, Andersson J, Tracey I (2014). Differential structural and resting state connectivity between insular subdivisions and other pain-related brain regions. Pain.

[7] Peyron R (2014). Functional imaging of pain. Biol Aujourdhui.

[8] Peyron R, Faillenot I, Pomares FB, Le Bars D, Garcia-Larrea L, Laurent B (2013). Mechanical allodynia in neuropathic pain. Where are the brain representations located? A positron emission tomography (PET) study. Eur J Pain.

[9] Qiu S, Zhang M, Liu Y, Guo Y, Zhao H, Song Q, Zhao M, Huganir RL (2014). GluA1 phosphorylation contributes to postsynaptic amplification of neuropathic pain in the insular cortex. J Neurosci.

[10] Liberati Giulia, Klöcker Anne, Algoet Maxime, Mulders Dounia, Maia Safronova Marta, Ferrao Santos Susana, Ribeiro Vaz José-Géraldo, Raftopoulos Christian, Mouraux André (2017). Gamma-Band Oscillations Preferential for Nociception can be Recorded in the Human Insula. Cerebral Cortex.

[11] Schulz E, May ES, Postorino M, Tiemann L, Nickel MM, Witkovsky V, Schmidt P, Gross J (2015). Prefrontal gamma oscillations encode tonic pain in humans. Cereb Cortex.

[12] Misra G, Wang WE, Archer DB, Roy A, Coombes SA (2017). Automated classification of pain perception using high-density electroencephalography data. J Neurophysiol.

[13] Garcia-Larrea L, Peyron R (2013). Pain matrices and neuropathic pain matrices: a review. Pain.

[14] Seminowicz DA, Davis KD (2006). Cortical responses to pain in healthy individuals depends on pain catastrophizing. Pain.

[15] Perlaki G, Orsi G, Schwarcz A, Bodi P, Plozer E, Biczo K, Aradi M, Doczi T (2015). Pain-related autonomic response is modulated by the medial prefrontal cortex: an ECG-fMRI study in men. J Neurol Sci.

[16] Karmann AJ, Maihofner C, Lautenbacher S, Sperling W, Kornhuber J, Kunz M (2016). The role of prefrontal inhibition in regulating facial expressions of pain: a repetitive transcranial magnetic stimulation study. J Pain.

[17] Fitzgerald M, Gruener G, Mtui E (2011) Clinical neuroanatomy and neuroscience. In., 6th edn. Elsevier Saunders

[18] An X, Bandler R, Ongur D, Price JL (1998). Prefrontal cortical projections to longitudinal columns in the midbrain periaqueductal gray in macaque monkeys. J Comp Neurol.

[19] Hardy SG, Leichnetz GR (1981). Cortical projections to the periaqueductal gray in the monkey: a retrograde and orthograde horseradish peroxidase study. Neuroscience letters.

[20] Bragin EO, Yeliseeva ZV, Vasilenko GF, Meizerov EE, Chuvin BT, Durinyan RA (1984). Cortical projections to the periaqueductal grey in the cat: a retrograde horseradish peroxidase study. Neuroscience letters.

[21] Carrive P, Morgan MM, Mai JK, Paxinos G (2011). Periaquiductal gray. The Human Nervous System. 2nd edn.

[22] Petrides M, Pandya DN, Mai JK, Paxinos G (2011). The Prefrontal Cortex. The Human Nervous System.

[23] Coulombe MA, Erpelding N, Kucyi A, Davis KD (2016). Intrinsic functional connectivity of periaqueductal gray subregions in humans. Hum Brain Mapp.

[24] Stein N, Sprenger C, Scholz J, Wiech K, Bingel U (2012). White matter integrity of the descending pain modulatory system is associated with interindividual differences in placebo analgesia. Pain.

[25] Hadjipavlou G, Dunckley P, Behrens TE, Tracey I (2006). Determining anatomical connectivities between cortical and brainstem pain processing regions in humans: a diffusion tensor imaging study in healthy controls. Pain.

[26] Butler RK, Nilsson-Todd L, Cleren C, Lena I, Garcia R, Finn DP (2011). Molecular and electrophysiological changes in the prefrontal cortex-amygdala-dorsal periaqueductal grey pathway during persistent pain state and fear-conditioned analgesia. Physiology & behavior.

[27] Mai JK, Paxinos G (2011) The human nervous system. Academic Press

[28] Tseng MT, Kong Y, Eippert F, Tracey I (2017). Determining the neural substrate for encoding a memory of human pain and the influence of anxiety. J Neurosci.

[29] Henderson LA, Peck CC, Petersen ET, Rae CD, Youssef AM, Reeves JM, Wilcox SL, Akhter R (2013). Chronic pain: lost inhibition?. J Neurosci.

[30] Jurik A, Auffenberg E, Klein S, Deussing JM, Schmid RM, Wotjak CT, Thoeringer CK (2015). Roles of prefrontal cortex and paraventricular thalamus in affective and mechanical components of visceral nociception. Pain.

[31] Cardoso-Cruz H, Sousa M, Vieira JB, Lima D, Galhardo V (2013). Prefrontal cortex and mediodorsal thalamus reduced connectivity is associated with spatial working memory impairment in rats with inflammatory pain. Pain.

[32] Lin HC, Huang YH, Chao TH, Lin WY, Sun WZ, Yen CT (2014). Gabapentin reverses central hypersensitivity and suppresses medial prefrontal cortical glucose metabolism in rats with neuropathic pain. Mol Pain.

[33] Simons LE, Pielech M, Erpelding N, Linnman C, Moulton E, Sava S, Lebel A, Serrano P (2014). The responsive amygdala: treatment-induced alterations in functional connectivity in pediatric complex regional pain syndrome. Pain.

[34] Cheriyan John, Kaushik Mahesh K., Ferreira Ashley N., Sheets Patrick L. (2016). Specific Targeting of the Basolateral Amygdala to Projectionally Defined Pyramidal Neurons in Prelimbic and Infralimbic Cortex. eneuro.

[35] Onozawa K, Yagasaki Y, Izawa Y, Abe H, Kawakami Y (2011). Amygdala-prefrontal pathways and the dopamine system affect nociceptive responses in the prefrontal cortex. BMC Neurosci.

[36] Ji G, Neugebauer V (2011). Pain-related deactivation of medial prefrontal cortical neurons involves mGluR1 and GABA(A) receptors. J Neurophysiol.

[37] Ji G, Sun H, Fu Y, Li Z, Pais-Vieira M, Galhardo V, Neugebauer V (2010). Cognitive impairment in pain through amygdala-driven prefrontal cortical deactivation. J Neurosci.

[38] Neugebauer V (2015). Amygdala pain mechanisms. Handb Exp Pharmacol.

[39] Kiritoshi Takaki, Neugebauer Volker (2018). Pathway-Specific Alterations of Cortico-Amygdala Transmission in an Arthritis Pain Model. ACS Chemical Neuroscience.

[40] Baliki MN, Petre B, Torbey S, Herrmann KM, Huang L, Schnitzer TJ, Fields HL, Apkarian AV (2012). Corticostriatal functional connectivity predicts transition to chronic back pain. Nat Neurosci.

[41] Apkarian AV, Baliki MN, Farmer MA (2013). Predicting transition to chronic pain. Curr Opin Neurol.

[42] Vachon-Presseau E, Tetreault P, Petre B, Huang L, Berger SE, Torbey S, Baria AT, Mansour AR (2016). Corticolimbic anatomical characteristics predetermine risk for chronic pain. Brain.

[43] Chang PC, Centeno MV, Procissi D, Baria A, Apkarian AV (2017). Brain activity for tactile allodynia: a longitudinal awake rat functional magnetic resonance imaging study tracking emergence of neuropathic pain. Pain.

[44] Huang X, Tang JS, Yuan B, Jia H (2001). Morphine applied to the ventrolateral orbital cortex produces a naloxone-reversible antinociception in the rat. Neurosci Lett.

[45] Qu CL, Tang JS, Jia H (2006). Involvement of GABAergic modulation of antinociception induced by morphine microinjected into the ventrolateral orbital cortex. Brain research.

[46] Tang JS, Qu CL, Huo FQ (2009). The thalamic nucleus submedius and ventrolateral orbital cortex are involved in nociceptive modulation: a novel pain modulation pathway. Progress in neurobiology.

[47] Sheng HY, Qu CL, Huo FQ, Du JQ, Tang JS (2009). D2-like but not D1-like dopamine receptors are involved in the ventrolateral orbital cortex-induced antinociception: a GABAergic modulation mechanism. Experimental neurology.

[48] Dang YH, Zhao Y, Xing B, Zhao XJ, Huo FQ, Tang JS, Qu CL, Chen T (2010). The role of dopamine receptors in ventrolateral orbital cortex-evoked anti-nociception in a rat model of neuropathic pain. Neuroscience.

[49] Sogabe S, Yagasaki Y, Onozawa K, Kawakami Y (2013). Mesocortical dopamine system modulates mechanical nociceptive responses recorded in the rat prefrontal cortex. BMC Neurosci.

[50] Wei L, Zhu YM, Zhang YX, Liang F, Jia H, Qu CL, Wang J, Tang JS (2016). Activation of alpha1 adrenoceptors in ventrolateral orbital cortex attenuates allodynia induced by spared nerve injury in rats. Neurochem Int.

[51] Millecamps M, Centeno MV, Berra HH, Rudick CN, Lavarello S, Tkatch T, Apkarian AV (2007). D-cycloserine reduces neuropathic pain behavior through limbic NMDA-mediated circuitry. Pain.

[52] Palazzo E, Luongo L, Guida F, Marabese I, Romano R, Iannotta M, Rossi F, D'Aniello A (2016). D-Aspartate drinking solution alleviates pain and cognitive impairment in neuropathic mice. Amino Acids.

[53] Sun Y, Liu K, Martinez E, Dale J, Huang D, Wang J (2017). AMPAkines and morphine provide complementary analgesia. Behav Brain Res.

[54] Zhang S, Tang JS, Yuan B, Jia H (1997). Involvement of the frontal ventrolateral orbital cortex in descending inhibition of nociception mediated by the periaqueductal gray in rats. Neuroscience letters.

[55] David-Pereira A, Puga S, Goncalves S, Amorim D, Silva C, Pertovaara A, Almeida A, Pinto-Ribeiro F (2016). Metabotropic glutamate 5 receptor in the infralimbic cortex contributes to descending pain facilitation in healthy and arthritic animals. Neuroscience.

[56] Hung KL, Wang SJ, Wang YC, Chiang TR, Wang CC (2014). Upregulation of presynaptic proteins and protein kinases associated with enhanced glutamate release from axonal terminals (synaptosomes) of the medial prefrontal cortex in rats with neuropathic pain. Pain.

[57] Zhao MG, Ko SW, Wu LJ, Toyoda H, Xu H, Quan J, Li J, Jia Y (2006). Enhanced presynaptic neurotransmitter release in the anterior cingulate cortex of mice with chronic pain. J Neurosci.

[58] Wang GQ, Cen C, Li C, Cao S, Wang N, Zhou Z, Liu XM, Xu Y (2015). Deactivation of excitatory neurons in the prelimbic cortex via Cdk5 promotes pain sensation and anxiety. Nat Commun.

[59] Chung G, Kim CY, Yun YC, Yoon SH, Kim MH, Kim YK, Kim SJ (2017). Upregulation of prefrontal metabotropic glutamate receptor 5 mediates neuropathic pain and negative mood symptoms after spinal nerve injury in rats. Sci Rep.

[60] Cordeiro Matos S, Zhang Z, Seguela P (2015). Peripheral neuropathy induces HCN channel dysfunction in pyramidal neurons of the medial prefrontal cortex. J Neurosci.

[61] Kiritoshi T, Sun H, Ren W, Stauffer SR, Lindsley CW, Conn PJ, Neugebauer V (2013). Modulation of pyramidal cell output in the medial prefrontal cortex by mGluR5 interacting with CB1. Neuropharmacology.

[62] Kiritoshi T, Ji G, Neugebauer V (2016). Rescue of Impaired mGluR5-driven endocannabinoid signaling restores prefrontal cortical output to inhibit pain in arthritic rats. J Neurosci.

[63] Kiritoshi T, Neugebauer V (2015). Group II mGluRs modulate baseline and arthritis pain-related synaptic transmission in the rat medial prefrontal cortex. Neuropharmacology.

[64] Luongo L, de Novellis V, Gatta L, Palazzo E, Vita D, Guida F, Giordano C, Siniscalco D (2013). Role of metabotropic glutamate receptor 1 in the basolateral amygdala-driven prefrontal cortical deactivation in inflammatory pain in the rat. Neuropharmacology.

[65] Zhang Z, Gadotti VM, Chen L, Souza IA, Stemkowski PL, Zamponi GW (2015). Role of prelimbic GABAergic circuits in sensory and emotional aspects of neuropathic pain. Cell Rep.

[66] Jones AK, Qi LY, Fujirawa T, Luthra SK, Ashburner J, Bloomfield P, Cunningham VJ, Itoh M (1991). In vivo distribution of opioid receptors in man in relation to the cortical projections of the medial and lateral pain systems measured with positron emission tomography. Neurosci Lett.

[67] Willoch F, Schindler F, Wester HJ, Empl M, Straube A, Schwaiger M, Conrad B, Tolle TR (2004). Central poststroke pain and reduced opioid receptor binding within pain processing circuitries: a [11C]diprenorphine PET study. Pain.

[68] Maarrawi J, Peyron R, Mertens P, Costes N, Magnin M, Sindou M, Laurent B, Garcia-Larrea L (2007). Differential brain opioid receptor availability in central and peripheral neuropathic pain. Pain.

[69] Zubieta JK, Smith YR, Bueller JA, Xu Y, Kilbourn MR, Jewett DM, Meyer CR, Koeppe RA (2001). Regional mu opioid receptor regulation of sensory and affective dimensions of pain. Science.

[70] Sprenger T, Berthele A, Platzer S, Boecker H, Tolle TR (2005). What to learn from in vivo opioidergic brain imaging?. Eur J Pain.

[71] Qiu YH, Wu XY, Xu H, Sackett D (2009). Neuroimaging study of placebo analgesia in humans. Neuroscience bulletin.

[72] Maarrawi J, Peyron R, Mertens P, Costes N, Magnin M, Sindou M, Laurent B, Garcia-Larrea L (2007). Motor cortex stimulation for pain control induces changes in the endogenous opioid system. Neurology.

[73] Zhao M, Wang JY, Jia H, Tang JS (2007). Roles of different subtypes of opioid receptors in mediating the ventrolateral orbital cortex opioid-induced inhibition of mirror-neuropathic pain in the rat. Neuroscience.

[74] Tejeda HA, Hanks AN, Scott L, Mejias-Aponte C, Hughes ZA, O'Donnell P (2015). Prefrontal cortical kappa opioid receptors attenuate responses to amygdala inputs. Neuropsychopharmacology.

[75] Palmisano M., Caputi F. F., Mercatelli D., Romualdi P., Candeletti S. (2018). Dynorphinergic system alterations in the corticostriatal circuitry of neuropathic mice support its role in the negative affective component of pain. Genes, Brain and Behavior.

[76] Finan PH, Goodin BR, Smith MT (2013). The association of sleep and pain: an update and a path forward. The journal of pain : official journal of the American Pain Society.

[77] Hoot MR, Sim-Selley LJ, Poklis JL, Abdullah RA, Scoggins KL, Selley DE, Dewey WL (2010). Chronic constriction injury reduces cannabinoid receptor 1 activity in the rostral anterior cingulate cortex of mice. Brain research.

[78] La Porta Carmen, Bura S. Andreea, Llorente-Onaindia Jone, Pastor Antoni, Navarrete Francisco, García-Gutiérrez María Salud, De la Torre Rafael, Manzanares Jorge, Monfort Jordi, Maldonado Rafael (2015). Role of the endocannabinoid system in the emotional manifestations of osteoarthritis pain. PAIN.

[79] Ji G, Neugebauer V (2014). CB1 augments mGluR5 function in medial prefrontal cortical neurons to inhibit amygdala hyperactivity in an arthritis pain model. Eur J Neurosci.

[80] Guida F, Luongo L, Marmo F, Romano R, Iannotta M, Napolitano F, Belardo C, Marabese I (2015). Palmitoylethanolamide reduces pain-related behaviors and restores glutamatergic synapses homeostasis in the medial prefrontal cortex of neuropathic mice. Mol Brain.

[81] de Novellis V, Vita D, Gatta L, Luongo L, Bellini G, De Chiaro M, Marabese I, Siniscalco D (2011). The blockade of the transient receptor potential vanilloid type 1 and fatty acid amide hydrolase decreases symptoms and central sequelae in the medial prefrontal cortex of neuropathic rats. Mol Pain.

[82] Ohara PT, Granato A, Moallem TM, Wang BR, Tillet Y, Jasmin L (2003). Dopaminergic input to GABAergic neurons in the rostral agranular insular cortex of the rat. J Neurocytol.

[83] Jarcho JM, Feier NA, Labus JS, Naliboff B, Smith SR, Hong JY, Colloca L, Tillisch K (2016). Placebo analgesia: self-report measures and preliminary evidence of cortical dopamine release associated with placebo response. Neuroimage Clin.

[84] Zhang Z, Cordeiro Matos S, Jego S, Adamantidis A, Seguela P (2013). Norepinephrine drives persistent activity in prefrontal cortex via synergistic alpha1 and alpha2 adrenoceptors. PLoS One.

[85] Taylor BK, Westlund KN (2017). The noradrenergic locus coeruleus as a chronic pain generator. J Neurosci Res.

[86] Alba-Delgado C, Llorca-Torralba M, Horrillo I, Ortega JE, Mico JA, Sanchez-Blazquez P, Meana JJ, Berrocoso E (2013). Chronic pain leads to concomitant noradrenergic impairment and mood disorders. Biol Psychiatry.

[87] Hirschberg S, Li Y, Randall A, Kremer EJ, Pickering AE (2017) Functional dichotomy in spinal- vs prefrontal-projecting locus coeruleus modules splits descending noradrenergic analgesia from ascending aversion and anxiety in rats. Elife 6. 10.7554/eLife.29808 10.7554/eLife.29808 PMC5653237 29027903

[88] Kaushal R, Taylor B, Jamal A, Zhang L, Ma F, Donahue R, Westlund K (2016). GABA-A receptor activity in the noradrenergic locus coeruleus drives trigeminal neuropathic pain in the rat; contribution of NAα1 receptors in the medial prefrontal cortex. Neuroscience.

[89] Cordeiro Matos S, Zamfir M, Longo G, Ribeiro-da-Silva A, Seguela P (2017) Noradrenergic fiber sprouting and altered transduction in neuropathic prefrontal cortex. Brain Struct Funct. 10.1007/s00429-017-1543-7 10.1007/s00429-017-1543-7 29094305

[90] Chu KL, Xu J, Frost J, Li L, Gomez E, Dart MJ, Jarvis MF, Meyer MD (2015). A selective alpha2 B adrenoceptor agonist (A-1262543) and duloxetine modulate nociceptive neurones in the medial prefrontal cortex, but not in the spinal cord of neuropathic rats. Eur J Pain.

[91] Radzicki D, Pollema-Mays SL, Sanz-Clemente A, Martina M (2017). Loss of M1 receptor dependent cholinergic excitation contributes to mPFC deactivation in neuropathic pain. J Neurosci.

[92] Naser Paul V., Kuner Rohini (2018). Molecular, Cellular and Circuit Basis of Cholinergic Modulation of Pain. Neuroscience.

[93] Grachev ID, Fredrickson BE, Apkarian AV (2000). Abnormal brain chemistry in chronic back pain: an in vivo proton magnetic resonance spectroscopy study. Pain.

[94] Grachev ID, Thomas PS, Ramachandran TS (2002). Decreased levels of N-acetylaspartate in dorsolateral prefrontal cortex in a case of intractable severe sympathetically mediated chronic pain (complex regional pain syndrome, type I). Brain Cogn.

[95] Palmisano M., Caputi F. F., Mercatelli D., Romualdi P., Candeletti S. (2018). Dynorphinergic system alterations in the corticostriatal circuitry of neuropathic mice support its role in the negative affective component of pain. Genes, Brain and Behavior.

[96] Yue L, Ma LY, Cui S, Liu FY, Yi M, Wan Y (2017). Brain-derived neurotrophic factor in the infralimbic cortex alleviates inflammatory pain. Neurosci Lett.

[97] Nir RR, Lev R, Moont R, Granovsky Y, Sprecher E, Yarnitsky D (2008). Neurophysiology of the cortical pain network: revisiting the role of S1 in subjective pain perception via standardized low-resolution brain electromagnetic tomography (sLORETA). J Pain.

[98] Nickel MM, May ES, Tiemann L, Schmidt P, Postorino M, Ta Dinh S, Gross J, Ploner M (2017). Brain oscillations differentially encode noxious stimulus intensity and pain intensity. Neuroimage.

[99] Benson S, Rebernik L, Wegner A, Kleine-Borgmann J, Engler H, Schlamann M, Forsting M, Schedlowski M (2015). Neural circuitry mediating inflammation-induced central pain amplification in human experimental endotoxemia. Brain Behav Immun.

[100] Karshikoff B, Jensen KB, Kosek E, Kalpouzos G, Soop A, Ingvar M, Olgart Hoglund C, Lekander M (2016). Why sickness hurts: a central mechanism for pain induced by peripheral inflammation. Brain Behav Immun.

[101] Brascher AK, Becker S, Hoeppli ME, Schweinhardt P (2016). Different brain circuitries mediating controllable and uncontrollable pain. J Neurosci.

[102] Kong J, Jensen K, Loiotile R, Cheetham A, Wey HY, Tan Y, Rosen B, Smoller JW (2013). Functional connectivity of the frontoparietal network predicts cognitive modulation of pain. Pain.

[103] Garza-Villarreal EA, Jiang Z, Vuust P, Alcauter S, Vase L, Pasaye EH, Cavazos-Rodriguez R, Brattico E (2015). Music reduces pain and increases resting state fMRI BOLD signal amplitude in the left angular gyrus in fibromyalgia patients. Front Psychol.

[104] Sakuma S, Inamoto K, Higuchi N, Ariji Y, Nakayama M, Izumi M (2014). Experimental pain in the gingiva and its impact on prefrontal cortical hemodynamics: a functional near-infrared spectroscopy study. Neurosci Lett.

[105] Elsenbruch S, Schmid J, Kullmann JS, Kattoor J, Theysohn N, Forsting M, Kotsis V (2014). Visceral sensitivity correlates with decreased regional gray matter volume in healthy volunteers: a voxel-based morphometry study. Pain.

[106] Ruscheweyh R, Kuhnel M, Filippopulos F, Blum B, Eggert T, Straube A (2014). Altered experimental pain perception after cerebellar infarction. Pain.

[107] Zunhammer M, Schweizer LM, Witte V, Harris RE, Bingel U, Schmidt-Wilcke T (2016). Combined glutamate and glutamine levels in pain-processing brain regions are associated with individual pain sensitivity. Pain.

[108] Singer T, Seymour B, O'Doherty J, Kaube H, Dolan RJ, Frith CD (2004). Empathy for pain involves the affective but not sensory components of pain. Science.

[109] Laneri D, Krach S, Paulus FM, Kanske P, Schuster V, Sommer J, Muller-Pinzler L (2017). Mindfulness meditation regulates anterior insula activity during empathy for social pain. Hum Brain Mapp.

[110] Danziger N, Faillenot I, Peyron R (2009). Can we share a pain we never felt? Neural correlates of empathy in patients with congenital insensitivity to pain. Neuron.

[111] Onoda K, Okamoto Y, Nakashima K, Nittono H, Ura M, Yamawaki S (2009). Decreased ventral anterior cingulate cortex activity is associated with reduced social pain during emotional support. Soc Neurosci.

[112] Poh KW, Yeo JF, Stohler CS, Ong WY (2012). Comprehensive gene expression profiling in the prefrontal cortex links immune activation and neutrophil infiltration to antinociception. The Journal of neuroscience : the official journal of the Society for Neuroscience.

[113] Pohl Kay-Wee, Yeol Jin-Fei, Ongl Wei-Yi (2011). MicroRNA changes in the mouse prefrontal cortex after inflammatory pain. European Journal of Pain.

[114] Devoize L, Alvarez P, Monconduit L, Dallel R (2011). Representation of dynamic mechanical allodynia in the ventral medial prefrontal cortex of trigeminal neuropathic rats. Eur J Pain.

[115] Fan XC, Fu S, Liu FY, Cui S, Yi M, Wan Y (2018). Hypersensitivity of prelimbic cortex neurons contributes to aggravated nociceptive responses in rats with experience of chronic inflammatory pain. Front Mol Neurosci.

[116] Murugan M, Ling EA, Kaur C (2013). Glutamate receptors in microglia. CNS Neurol Disord Drug Targets.

[117] Zhang F, Vadakkan KI, Kim SS, Wu LJ, Shang Y, Zhuo M (2008). Selective activation of microglia in spinal cord but not higher cortical regions following nerve injury in adult mouse. Mol Pain.

[118] del Rey A, Apkarian AV, Martina M, Besedovsky HO (2012). Chronic neuropathic pain-like behavior and brain-borne IL-1beta. Ann N Y Acad Sci.

[119] Shao Q, Li Y, Wang Q, Zhao J (2015). IL-10 and IL-1beta mediate neuropathic-pain like behavior in the ventrolateral orbital cortex. Neurochem Res.

[120] Gonzalez-Sepulveda M, Pozo OJ, Marcos J, Valverde O (2016). Chronic pain causes a persistent anxiety state leading to increased ethanol intake in CD1 mice. J Psychopharmacol.

[121] Tramullas M, Finger BC, Moloney RD, Golubeva AV, Moloney G, Dinan TG, Cryan JF (2014). Toll-like receptor 4 regulates chronic stress-induced visceral pain in mice. Biol Psychiatry.

[122] Tramullas M, Finger BC, Dinan TG, Cryan JF (2016). Obesity takes Its toll on visceral pain: high-fat diet induces toll-like receptor 4-dependent visceral hypersensitivity. PLoS One.

[123] Boadas-Vaello Pere, Homs Judit, Reina Francisco, Carrera Ana, Verdú Enrique (2017). Neuroplasticity of Supraspinal Structures Associated with Pathological Pain. The Anatomical Record.

[124] Chu Sin Chung P, Panigada T, Cardis R, Decosterd I, Gosselin RD (2017). Peripheral nerve injury induces a transitory microglial reaction in the rat infralimbic cortex. Neurosci Lett.

[125] Gui Wen-Shan, Wei Xiao, Mai Chun-Lin, Murugan Madhuvika, Wu Long-Jun, Xin Wen-Jun, Zhou Li-Jun, Liu Xian-Guo (2016). Interleukin-1β overproduction is a common cause for neuropathic pain, memory deficit, and depression following peripheral nerve injury in rodents. Molecular Pain.

[126] Burke Nikita N., Kerr Daniel M., Moriarty Orla, Finn David P., Roche Michelle (2014). Minocycline modulates neuropathic pain behaviour and cortical M1–M2 microglial gene expression in a rat model of depression. Brain, Behavior, and Immunity.

[127] Xu N, Tang XH, Pan W, Xie ZM, Zhang GF, Ji MH, Yang JJ, Zhou MT (2017). Spared nerve injury increases the expression of microglia M1 markers in the prefrontal cortex of rats and provokes depression-like behaviors. Front Neurosci.

[128] Marcello L, Cavaliere C, Colangelo AM, Bianco MR, Cirillo G, Alberghina L, Papa M (2013). Remodelling of supraspinal neuroglial network in neuropathic pain is featured by a reactive gliosis of the nociceptive amygdala. Eur J Pain.

[129] Jia D, Gao GD, Liu Y, He SM, Zhang XN, Zhang YF, Zhao MG (2007). TNF-alpha involves in altered prefrontal synaptic transmission in mice with persistent inflammatory pain. Neurosci Lett.

[130] Cui GB, An JZ, Zhang N, Zhao MG, Liu SB, Yi J (2012). Elevated interleukin-8 enhances prefrontal synaptic transmission in mice with persistent inflammatory pain. Mol Pain.

[131] Wu XB, Liang B, Gao YJ (2016). The increase of intrinsic excitability of layer V pyramidal cells in the prelimbic medial prefrontal cortex of adult mice after peripheral inflammation. Neurosci Lett.

[132] Riego G, Redondo A, Leanez S, Pol O (2018). Mechanism implicated in the anti-allodynic and anti-hyperalgesic effects induced by the activation of heme oxygenase 1/carbon monoxide signaling pathway in the central nervous system of mice with neuropathic pain. Biochem Pharmacol.

[133] Li Z, Wei H, Piirainen S, Chen Z, Kalso E, Pertovaara A, Tian L (2016). Spinal versus brain microglial and macrophage activation traits determine the differential neuroinflammatory responses and analgesic effect of minocycline in chronic neuropathic pain. Brain Behav Immun.

[134] Panigada T, Gosselin RD (2011). Behavioural alteration in chronic pain: are brain glia involved?. Med Hypotheses.

[135] Fiore NT, Austin PJ (2016). Are the emergence of affective disturbances in neuropathic pain states contingent on supraspinal neuroinflammation?. Brain Behav Immun.

[136] Yuan CongHu, Shi HaiCun, Pan PingLei, Dai ZhenYu, Zhong JianGuo, Ma HaiRong, Sheng LiQin (2017). Gray Matter Abnormalities Associated With Chronic Back Pain. The Clinical Journal of Pain.

[137] Apkarian AV, Sosa Y, Sonty S, Levy RM, Harden RN, Parrish TB, Gitelman DR (2004). Chronic back pain is associated with decreased prefrontal and thalamic gray matter density. The Journal of neuroscience : the official journal of the Society for Neuroscience.

[138] Ivo R, Nicklas A, Dargel J, Sobottke R, Delank KS, Eysel P, Weber B (2013). Brain structural and psychometric alterations in chronic low back pain. Eur Spine J.

[139] Luchtmann M, Steinecke Y, Baecke S, Lutzkendorf R, Bernarding J, Kohl J, Jollenbeck B, Tempelmann C (2014). Structural brain alterations in patients with lumbar disc herniation: a preliminary study. PLoS One.

[140] Fritz HC, McAuley JH, Wittfeld K, Hegenscheid K, Schmidt CO, Langner S, Lotze M (2016). Chronic back pain is associated with decreased prefrontal and anterior insular gray matter: results from a population-based cohort study. J Pain.

[141] Wu Q, Inman RD, Davis KD (2013). Neuropathic pain in ankylosing spondylitis: a psychophysics and brain imaging study. Arthritis Rheum.

[142] Geha PY, Baliki MN, Harden RN, Bauer WR, Parrish TB, Apkarian AV (2008). The brain in chronic CRPS pain: abnormal gray-white matter interactions in emotional and autonomic regions. Neuron.

[143] Barad MJ, Ueno T, Younger J, Chatterjee N, Mackey S (2014). Complex regional pain syndrome is associated with structural abnormalities in pain-related regions of the human brain. J Pain.

[144] Kuchinad A, Schweinhardt P, Seminowicz DA, Wood PB, Chizh BA, Bushnell MC (2007). Accelerated brain gray matter loss in fibromyalgia patients: premature aging of the brain?. The Journal of neuroscience : the official journal of the Society for Neuroscience.

[145] Niddam DM, Lee SH, Su YT, Chan RC (2017). Brain structural changes in patients with chronic myofascial pain. Eur J Pain.

[146] Valet M, Gundel H, Sprenger T, Sorg C, Muhlau M, Zimmer C, Henningsen P, Tolle TR (2009). Patients with pain disorder show gray-matter loss in pain-processing structures: a voxel-based morphometric study. Psychosom Med.

[147] Gustin SM, Wrigley PJ, Siddall PJ, Henderson LA (2010). Brain anatomy changes associated with persistent neuropathic pain following spinal cord injury. Cereb Cortex.

[148] Yoon EJ, Kim YK, Shin HI, Lee Y, Kim SE (2013). Cortical and white matter alterations in patients with neuropathic pain after spinal cord injury. Brain Res.

[149] Krause T, Asseyer S, Taskin B, Floel A, Witte AV, Mueller K, Fiebach JB, Villringer K (2016). The cortical signature of central poststroke pain: gray matter decreases in somatosensory, insular, and prefrontal cortices. Cereb Cortex.

[150] van der Schaaf ME, De Lange FP, Schmits IC, Geurts DE, Roelofs K, van der Meer JW, Toni I, Knoop H (2017). Prefrontal structure varies as a function of pain symptoms in chronic fatigue syndrome. Biol Psychiatry.

[151] Obermann M, Rodriguez-Raecke R, Naegel S, Holle D, Mueller D, Yoon MS, Theysohn N, Blex S (2013). Gray matter volume reduction reflects chronic pain in trigeminal neuralgia. Neuroimage.

[152] Wang Yuan, Cao Dong-yuan, Remeniuk Bethany, Krimmel Samuel, Seminowicz David A., Zhang Ming (2017). Altered brain structure and function associated with sensory and affective components of classic trigeminal neuralgia. PAIN.

[153] Gerstner G, Ichesco E, Quintero A, Schmidt-Wilcke T (2011). Changes in regional gray and white matter volume in patients with myofascial-type temporomandibular disorders: a voxel-based morphometry study. Journal of orofacial pain.

[154] Khan SA, Keaser ML, Meiller TF, Seminowicz DA (2014). Altered structure and function in the hippocampus and medial prefrontal cortex in patients with burning mouth syndrome. Pain.

[155] Vartiainen N, Kallio-Laine K, Hlushchuk Y, Kirveskari E, Seppanen M, Autti H, Jousmaki V, Forss N (2009). Changes in brain function and morphology in patients with recurring herpes simplex virus infections and chronic pain. Pain.

[156] Liu Peng, Wang Geliang, Zeng Fang, Liu Yanfei, Fan Yingying, Wei Ying, Qin Wei, Calhoun Vince D. (2017). Abnormal brain structure implicated in patients with functional dyspepsia. Brain Imaging and Behavior.

[157] Frøkjær Jens Brøndum, Bouwense Stefan A.W., Olesen Søren Schou, Lundager Flemming H., Eskildsen Simon F., van Goor Harry, Wilder–Smith Oliver H.G., Drewes Asbjørn Mohr (2012). Reduced Cortical Thickness of Brain Areas Involved in Pain Processing in Patients With Chronic Pancreatitis. Clinical Gastroenterology and Hepatology.

[158] Seminowicz DA, Labus JS, Bueller JA, Tillisch K, Naliboff BD, Bushnell MC, Mayer EA (2010). Regional gray matter density changes in brains of patients with irritable bowel syndrome. Gastroenterology.

[159] Chua CS, Bai CH, Shiao CY, Hsu CY, Cheng CW, Yang KC, Chiu HW, Hsu JL (2017). Negative correlation of cortical thickness with the severity and duration of abdominal pain in Asian women with irritable bowel syndrome. PLoS One.

[160] Hubbard CS, Becerra L, Heinz N, Ludwick A, Rasooly T, Wu R, Johnson A, Schechter NL (2016). Abdominal pain, the adolescent and altered brain structure and function. PLoS One.

[161] Piche M, Chen JI, Roy M, Poitras P, Bouin M, Rainville P (2013). Thicker posterior insula is associated with disease duration in women with irritable bowel syndrome (IBS) whereas thicker orbitofrontal cortex predicts reduced pain inhibition in both IBS patients and controls. J Pain.

[162] Gustin SM, McKay JG, Petersen ET, Peck CC, Murray GM, Henderson LA (2014). Subtle alterations in brain anatomy may change an individual's personality in chronic pain. PLoS One.

[163] Xie P, Qin B, Song G, Zhang Y, Cao S, Yu J, Wu J, Wang J (2016). Microstructural abnormalities were found in brain gray matter from patients with chronic myofascial pain. Front Neuroanat.

[164] Metz AE, Yau HJ, Centeno MV, Apkarian AV, Martina M (2009). Morphological and functional reorganization of rat medial prefrontal cortex in neuropathic pain. Proc Natl Acad Sci U S A.

[165] Kelly CJ, Huang M, Meltzer H, Martina M (2016). Reduced glutamatergic currents and dendritic branching of layer 5 pyramidal cells contribute to medial prefrontal cortex deactivation in a rat model of neuropathic pain. Front Cell Neurosci.

[166] Nakamura Y, Nojiri K, Yoshihara H, Takahata T, Honda-Takahashi K, Kubo S, Sakatsume K, Kato H (2014). Significant differences of brain blood flow in patients with chronic low back pain and acute low back pain detected by brain SPECT. J Orthop Sci.

[167] Lim M, Kim JS, Kim DJ, Chung CK (2016). Increased low- and high-frequency oscillatory activity in the prefrontal cortex of fibromyalgia patients. Front Hum Neurosci.

[168] Fallon N, Chiu Y, Nurmikko T, Stancak A (2018). Altered theta oscillations in resting EEG of fibromyalgia syndrome patients. Eur J Pain.

[169] Lev R, Granovsky Y, Yarnitsky D (2013). Enhanced pain expectation in migraine: EEG-based evidence for impaired prefrontal function. Headache.

[170] Burgmer M, Pfleiderer B, Maihofner C, Gaubitz M, Wessolleck E, Heuft G, Pogatzki-Zahn E (2012). Cerebral mechanisms of experimental hyperalgesia in fibromyalgia. Eur J Pain.

[171] Lev R, Granovsky Y, Yarnitsky D (2010). Orbitofrontal disinhibition of pain in migraine with aura: an interictal EEG-mapping study. Cephalalgia.

[172] Derbyshire SW, Jones AK, Devani P, Friston KJ, Feinmann C, Harris M, Pearce S, Watson JD (1994). Cerebral responses to pain in patients with atypical facial pain measured by positron emission tomography. Journal of neurology, neurosurgery, and psychiatry.

[173] Dale J, Zhou H, Zhang Q, Martinez E, Hu S, Liu K, Urien L, Chen Z (2018). Scaling up cortical control inhibits pain. Cell Rep.

[174] Cheriyan John, Sheets Patrick L. (2018). Altered Excitability and Local Connectivity of mPFC-PAG Neurons in a Mouse Model of Neuropathic Pain. The Journal of Neuroscience.

[175] Yu R, Gollub RL, Spaeth R, Napadow V, Wasan A, Kong J (2014). Disrupted functional connectivity of the periaqueductal gray in chronic low back pain. Neuroimage Clin.

[176] Baliki MN, Mansour AR, Baria AT, Apkarian AV (2014). Functional reorganization of the default mode network across chronic pain conditions. PLoS One.

[177] Mutso AA, Petre B, Huang L, Baliki MN, Torbey S, Herrmann KM, Schnitzer TJ, Apkarian AV (2014). Reorganization of hippocampal functional connectivity with transition to chronic back pain. J Neurophysiol.

[178] Flodin P, Martinsen S, Lofgren M, Bileviciute-Ljungar I, Kosek E, Fransson P (2014). Fibromyalgia is associated with decreased connectivity between pain- and sensorimotor brain areas. Brain Connect.

[179] Weng Y, Qi R, Liu C, Ke J, Xu Q, Wang F, Zhang LJ, Lu GM (2016) Disrupted functional connectivity density in irritable bowel syndrome patients. Brain imaging and behavior:1-11 10.1007/s11682-016-9653-z 27848148

[180] Wei SY, Chao HT, Tu CH, Li WC, Low I, Chuang CY, Chen LF, Hsieh JC (2016). Changes in functional connectivity of pain modulatory systems in women with primary dysmenorrhea. Pain.

[181] Gao Q, Xu F, Jiang C, Chen Z, Chen H, Liao H, Zhao L (2016). Decreased functional connectivity density in pain-related brain regions of female migraine patients without aura. Brain Res.

[182] Solstrand Dahlberg L, Linnman CN, Lee D, Burstein R, Becerra L, Borsook D (2018). Responsivity of periaqueductal gray connectivity is related to headache frequency in episodic migraine. Front Neurol.

[183] Oosterman JM, van Harten B, Weinstein HC, Scheltens P, Scherder EJ (2006). Pain intensity and pain affect in relation to white matter changes. Pain.

[184] Dun WH, Yang J, Yang L, Ding D, Ma XY, Liang FL, von Deneen KM, Ma SH (2017). Abnormal structure and functional connectivity of the anterior insula at pain-free periovulation is associated with perceived pain during menstruation. Brain Imaging Behav.

[185] Coppola G, Di Renzo A, Tinelli E, Di Lorenzo C, Scapeccia M, Parisi V, Serrao M, Evangelista M (2018). Resting state connectivity between default mode network and insula encodes acute migraine headache. Cephalalgia.

[186] Cottam WJ, Iwabuchi SJ, Drabek MM, Reckziegel D, Auer DP (2018). Altered connectivity of the right anterior insula drives the pain connectome changes in chronic knee osteoarthritis. Pain.

[187] Hauck M, Schroder S, Meyer-Hamme G, Lorenz J, Friedrichs S, Nolte G, Gerloff C, Engel AK (2017). Acupuncture analgesia involves modulation of pain-induced gamma oscillations and cortical network connectivity. Scientific reports.

[188] As-Sanie S, Kim J, Schmidt-Wilcke T, Sundgren PC, Clauw DJ, Napadow V, Harris RE (2016). Functional connectivity is associated with altered brain chemistry in women with endometriosis-associated chronic pelvic pain. J Pain.

[189] Flodin P, Martinsen S, Altawil R, Waldheim E, Lampa J, Kosek E, Fransson P (2016). Intrinsic brain connectivity in chronic pain: a resting-state fMRI study in patients with rheumatoid arthritis. Front Hum Neurosci.

[190] Moayedi M, Weissman-Fogel I, Salomons TV, Crawley AP, Goldberg MB, Freeman BV, Tenenbaum HC, Davis KD (2012). White matter brain and trigeminal nerve abnormalities in temporomandibular disorder. Pain.

[191] Bishop James H., Shpaner Marina, Kubicki Antoni, Clements Sarah, Watts Richard, Naylor Magdalena R. (2018). Structural network differences in chronic muskuloskeletal pain: Beyond fractional anisotropy. NeuroImage.

[192] Kucyi A, Moayedi M, Weissman-Fogel I, Goldberg MB, Freeman BV, Tenenbaum HC, Davis KD (2014). Enhanced medial prefrontal-default mode network functional connectivity in chronic pain and its association with pain rumination. J Neurosci.

[193] Zhao X, Xu M, Jorgenson K, Kong J (2017). Neurochemical changes in patients with chronic low back pain detected by proton magnetic resonance spectroscopy: a systematic review. Neuroimage Clin.

[194] Kosek E, Martinsen S, Gerdle B, Mannerkorpi K, Lofgren M, Bileviciute-Ljungar I, Fransson P, Schalling M (2016). The translocator protein gene is associated with symptom severity and cerebral pain processing in fibromyalgia. Brain Behav Immun.

[195] Alvarado S, Tajerian M, Millecamps M, Suderman M, Stone LS, Szyf M (2013). Peripheral nerve injury is accompanied by chronic transcriptome-wide changes in the mouse prefrontal cortex. Molecular pain.

[196] Tajerian M, Alvarado S, Millecamps M, Vachon P, Crosby C, Bushnell MC, Szyf M, Stone LS (2013). Peripheral nerve injury is associated with chronic, reversible changes in global DNA methylation in the mouse prefrontal cortex. PLoS One.

[197] Alvarado S, Tajerian M, Suderman M, Machnes Z, Pierfelice S, Millecamps M, Stone LS, Szyf M (2015). An epigenetic hypothesis for the genomic memory of pain. Front Cell Neurosci.

[198] Massart R, Dymov S, Millecamps M, Suderman M, Gregoire S, Koenigs K, Alvarado S, Tajerian M (2016). Overlapping signatures of chronic pain in the DNA methylation landscape of prefrontal cortex and peripheral T cells. Sci Rep.

[199] Yan S, Kentner AC (2017). Mechanical allodynia corresponds to Oprm1 downregulation within the descending pain network of male and female rats exposed to neonatal immune challenge. Brain Behav Immun.

[200] Sanada LS, Sato KL, Machado NL, Carmo Ede C, Sluka KA, Fazan VP (2014). Cortex glial cells activation, associated with lowered mechanical thresholds and motor dysfunction, persists into adulthood after neonatal pain. Int J Dev Neurosci.

[201] Martinez E, Lin HH, Zhou H, Dale J, Liu K, Wang J (2017). Corticostriatal regulation of acute pain. Front Cell Neurosci.

[202] Lee M, Manders TR, Eberle SE, Su C, D'Amour J, Yang R, Lin HY, Deisseroth K (2015). Activation of corticostriatal circuitry relieves chronic neuropathic pain. J Neurosci.

[203] Mansour AR, Baliki MN, Huang L, Torbey S, Herrmann KM, Schnitzer TJ, Apkarian AV (2013). Brain white matter structural properties predict transition to chronic pain. Pain.

[204] Bilbao Ainhoa, Falfán-Melgoza Claudia, Leixner Sarah, Becker Robert, Singaravelu Sathish Kumar, Sack Markus, Sartorius Alexander, Spanagel Rainer, Weber-Fahr Wolfgang (2018). Longitudinal Structural and Functional Brain Network Alterations in a Mouse Model of Neuropathic Pain. Neuroscience.

[205] Blanchet Pierre J., Brefel-Courbon Christine (2018). Chronic pain and pain processing in Parkinson's disease. Progress in Neuro-Psychopharmacology and Biological Psychiatry.

[206] Forkmann Katarina, Grashorn Wiebke, Schmidt Katharina, Fründt Odette, Buhmann Carsten, Bingel Ulrike (2017). Altered neural responses to heat pain in drug-naive patients with Parkinson disease. PAIN.

[207] Petre B, Torbey S, Griffith JW, De Oliveira G, Herrmann K, Mansour A, Baria AT, Baliki MN (2015). Smoking increases risk of pain chronification through shared corticostriatal circuitry. Hum Brain Mapp.

[208] Volkow ND, Morales M (2015). The brain on drugs: from reward to addiction. Cell.

[209] Becker S, Gandhi W, Pomares F, Wager TD, Schweinhardt P (2017). Orbitofrontal cortex mediates pain inhibition by monetary reward. Soc Cogn Affect Neurosci.

[210] Leitl MD, Onvani S, Bowers MS, Cheng K, Rice KC, Carlezon WA, Banks ML, Negus SS (2014). Pain-related depression of the mesolimbic dopamine system in rats: expression, blockade by analgesics, and role of endogenous kappa-opioids. Neuropsychopharmacology.

[211] Elman I, Borsook D, Volkow ND (2013). Pain and suicidality: insights from reward and addiction neuroscience. Progress in neurobiology.

[212] Percheron G, Mai JK, Paxinos G (2011). Thalamus. The Human Nervous System. 2nd edn.

[213] Roberts TS, Akert K (1963). Insular and opercular cortex and its thalamic projection in Macaca mulatta. Schweiz Arch Neurol Neurochir Psychiatr.

[214] Wirth FP (1973). Insular-diencephalic connections in the Macaque. J Comp Neurol.

[215] Mufson EJ, Mesulam MM (1984). Thalamic connections of the insula in the rhesus monkey and comments on the paralimbic connectivity of the medial pulvinar nucleus. J Comp Neurol.

[216] Haber SN, Gdowski MJ, Mai JK, Paxinos G (2011). The basal ganglia. The Human Nervous System. 2nd edn.

[217] Martin L, Borckardt JJ, Reeves ST, Frohman H, Beam W, Nahas Z, Johnson K, Younger J (2013). A pilot functional MRI study of the effects of prefrontal rTMS on pain perception. Pain Med.

[218] Boggio PS, Zaghi S, Lopes M, Fregni F (2008). Modulatory effects of anodal transcranial direct current stimulation on perception and pain thresholds in healthy volunteers. European journal of neurology : the official journal of the European Federation of Neurological Societies.

[219] Dockery CA, Hueckel-Weng R, Birbaumer N, Plewnia C (2009). Enhancement of planning ability by transcranial direct current stimulation. J Neurosci.

[220] Allan C, Kalu UG, Sexton CE, Ebmeier KP (2012). Transcranial stimulation in depression. Br J Psychiatry.

[221] Stagg CJ, Lin RL, Mezue M, Segerdahl A, Kong Y, Xie J, Tracey I (2013). Widespread modulation of cerebral perfusion induced during and after transcranial direct current stimulation applied to the left dorsolateral prefrontal cortex. J Neurosci.

[222] Lopez-Sola M, Pujol J, Hernandez-Ribas R, Harrison BJ, Contreras-Rodriguez O, Soriano-Mas C, Deus J, Ortiz H (2010). Effects of duloxetine treatment on brain response to painful stimulation in major depressive disorder. Neuropsychopharmacology.

[223] Pariente J, White P, Frackowiak RS, Lewith G (2005). Expectancy and belief modulate the neuronal substrates of pain treated by acupuncture. Neuroimage.

[224] Bai L, Tian J, Zhong C, Xue T, You Y, Liu Z, Chen P, Gong Q (2010). Acupuncture modulates temporal neural responses in wide brain networks: evidence from fMRI study. Molecular pain.

[225] Kocyigit F, Akalin E, Gezer NS, Orbay O, Kocyigit A, Ada E (2012). Functional magnetic resonance imaging of the effects of low-frequency transcutaneous electrical nerve stimulation on central pain modulation: a double-blind, placebo-controlled trial. The Clinical journal of pain.

[226] Jensen KB, Kosek E, Wicksell R, Kemani M, Olsson G, Merle JV, Kadetoff D, Ingvar M (2012). Cognitive behavioral therapy increases pain-evoked activation of the prefrontal cortex in patients with fibromyalgia. Pain.

[227] Gard T, Holzel BK, Sack AT, Hempel H, Lazar SW, Vaitl D, Ott U (2012). Pain attenuation through mindfulness is associated with decreased cognitive control and increased sensory processing in the brain. Cereb Cortex.

[228] Zeidan F, Emerson NM, Farris SR, Ray JN, Jung Y, McHaffie JG, Coghill RC (2015). Mindfulness meditation-based pain relief employs different neural mechanisms than placebo and sham mindfulness meditation-induced analgesia. J Neurosci.

[229] Bilevicius Elena, Kolesar Tiffany, Kornelsen Jennifer (2016). Altered Neural Activity Associated with Mindfulness during Nociception: A Systematic Review of Functional MRI. Brain Sciences.

[230] Dobek CE, Beynon ME, Bosma RL, Stroman PW (2014). Music modulation of pain perception and pain-related activity in the brain, brain stem, and spinal cord: a functional magnetic resonance imaging study. J Pain.

[231] Ellingson Laura, Stegner Aaron, Schwabacher Isaac, Koltyn Kelli, Cook Dane (2016). Exercise Strengthens Central Nervous System Modulation of Pain in Fibromyalgia. Brain Sciences.

[232] Eisenberger NI, Master SL, Inagaki TK, Taylor SE, Shirinyan D, Lieberman MD, Naliboff BD (2011). Attachment figures activate a safety signal-related neural region and reduce pain experience. Proc Natl Acad Sci U S A.

[233] Brown CA, Jones AK (2010). Meditation experience predicts less negative appraisal of pain: electrophysiological evidence for the involvement of anticipatory neural responses. Pain.

[234] Orme-Johnson DW, Schneider RH, Son YD, Nidich S, Cho ZH (2006). Neuroimaging of meditation's effect on brain reactivity to pain. Neuroreport.

[235] Grant JA, Courtemanche J, Rainville P (2011). A non-elaborative mental stance and decoupling of executive and pain-related cortices predicts low pain sensitivity in Zen meditators. Pain.

[236] Elmholdt EM, Skewes J, Dietz M, Moller A, Jensen MS, Roepstorff A, Wiech K, Jensen TS (2017). Reduced pain sensation and reduced BOLD signal in parietofrontal networks during religious prayer. Front Hum Neurosci.

[237] Graff-Guerrero A, Gonzalez-Olvera J, Fresan A, Gomez-Martin D, Mendez-Nunez JC, Pellicer F (2005). Repetitive transcranial magnetic stimulation of dorsolateral prefrontal cortex increases tolerance to human experimental pain. Brain Res Cogn Brain Res.

[238] Brighina F, De Tommaso M, Giglia F, Scalia S, Cosentino G, Puma A, Panetta M, Giglia G (2011). Modulation of pain perception by transcranial magnetic stimulation of left prefrontal cortex. The journal of headache and pain.

[239] Taylor JJ, Borckardt JJ, Canterberry M, Li X, Hanlon CA, Brown TR, George MS (2013). Naloxone-reversible modulation of pain circuitry by left prefrontal rTMS. Neuropsychopharmacology.

[240] Short EB, Borckardt JJ, Anderson BS, Frohman H, Beam W, Reeves ST, George MS (2011). Ten sessions of adjunctive left prefrontal rTMS significantly reduces fibromyalgia pain: a randomized, controlled pilot study. Pain.

[241] Brighina F, Piazza A, Vitello G, Aloisio A, Palermo A, Daniele O, Fierro B (2004). rTMS of the prefrontal cortex in the treatment of chronic migraine: a pilot study. Journal of the neurological sciences.

[242] Leung Albert, Metzger-Smith Valerie, He Yifan, Cordero James, Ehlert Brandon, Song David, Lin Lisa, Shahrokh Golshan, Tsai Alice, Vaninetti Michael, Rutledge Thomas, Polston Greg, Sheu Robert, Lee Roland (2017). Left Dorsolateral Prefrontal Cortex rTMS in Alleviating MTBI Related Headaches and Depressive Symptoms. Neuromodulation: Technology at the Neural Interface.

[243] Conforto Adriana B, Amaro Edson, Gonçalves André L, Mercante Juliane PP, Guendler Vera Z, Ferreira Josione R, Kirschner Clara CFB, Peres Mario FP (2013). Randomized, proof-of-principle clinical trial of active transcranial magnetic stimulation in chronic migraine. Cephalalgia.

[244] Umezaki Y, Badran BW, DeVries WH, Moss J, Gonzales T, George MS (2016). The efficacy of daily prefrontal repetitive transcranial magnetic stimulation (rTMS) for burning mouth syndrome (BMS): a randomized controlled single-blind study. Brain Stimul.

[245] Borckardt JJ, Reeves ST, Weinstein M, Smith AR, Shelley N, Kozel FA, Nahas Z, Byrne KT (2008). Significant analgesic effects of one session of postoperative left prefrontal cortex repetitive transcranial magnetic stimulation: a replication study. Brain stimulation.

[246] Borckardt JJ, Weinstein M, Reeves ST, Kozel FA, Nahas Z, Smith AR, Byrne TK, Morgan K (2006). Postoperative left prefrontal repetitive transcranial magnetic stimulation reduces patient-controlled analgesia use. Anesthesiology.

[247] Taylor SJ, Mann TS, Henry PJ (2012). Influence of influenza A infection on capsaicin-induced responses in murine airways. The Journal of pharmacology and experimental therapeutics.

[248] Lamusuo S., Hirvonen J., Lindholm P., Martikainen I. K., Hagelberg N., Parkkola R., Taiminen T., Hietala J., Helin S., Virtanen A., Pertovaara A., Jääskeläinen S.K. (2017). Neurotransmitters behind pain relief with transcranial magnetic stimulation - positron emission tomography evidence for release of endogenous opioids. European Journal of Pain.

[249] de Andrade DC, Mhalla A, Adam F, Texeira MJ, Bouhassira D (2011). Neuropharmacological basis of rTMS-induced analgesia: the role of endogenous opioids. Pain.

[250] Ciampi de Andrade D, Mhalla A, Adam F, Texeira MJ, Bouhassira D (2014). Repetitive transcranial magnetic stimulation induced analgesia depends on N-methyl-D-aspartate glutamate receptors. Pain.

[251] Lee LHW, Tan CH, Lo YL, Farooqui AA, Shui G, Wenk MR, Ong WY (2011). Brain lipid changes after repetitive transcranial magnetic stimulation: potential links to therapeutic effects?. Metabolomics.

[252] Shalini SM, Herr DR, Ong WY (2017). The analgesic and anxiolytic effect of Souvenaid, a novel nutraceutical, is mediated by Alox15 activity in the prefrontal cortex. Mol Neurobiol.

[253] Shalini Suku-Maran, Ho Christabel Fung-Yih, Ng Yee-Kong, Tong Jie-Xin, Ong Eng-Shi, Herr Deron R., Dawe Gavin S., Ong Wei-Yi (2017). Distribution of Alox15 in the Rat Brain and Its Role in Prefrontal Cortical Resolvin D1 Formation and Spatial Working Memory. Molecular Neurobiology.

[254] Moisset X, de Andrade DC, Bouhassira D (2016). From pulses to pain relief: an update on the mechanisms of rTMS-induced analgesic effects. Eur J Pain.

[255] Maeoka H, Matsuo A, Hiyamizu M, Morioka S, Ando H (2012). Influence of transcranial direct current stimulation of the dorsolateral prefrontal cortex on pain related emotions: a study using electroencephalographic power spectrum analysis. Neuroscience letters.

[256] Lin RL, Douaud G, Filippini N, Okell TW, Stagg CJ, Tracey I (2017). Structural connectivity variances underlie functional and behavioral changes during pain relief induced by neuromodulation. Sci Rep.

[257] Glaser J, Reeves ST, Stoll WD, Epperson TI, Hilbert M, Madan A, George MS, Borckardt JJ (2016). Motor/prefrontal transcranial direct current stimulation (tDCS) following lumbar surgery reduces postoperative analgesia use. Spine (Phila Pa 1976).

[258] Borckardt JJ, Reeves ST, Robinson SM, May JT, Epperson TI, Gunselman RJ, Schutte HD, Demos HA (2013). Transcranial direct current stimulation (tDCS) reduces postsurgical opioid consumption in total knee arthroplasty (TKA). The Clinical journal of pain.

[259] Choi YH, Jung SJ, Lee CH, Lee SU (2014). Additional effects of transcranial direct-current stimulation and trigger-point injection for treatment of myofascial pain syndrome: a pilot study with randomized, single-blinded trial. Journal of alternative and complementary medicine.

[260] Ayache SS, Palm U, Chalah MA, Al-Ani T, Brignol A, Abdellaoui M, Dimitri D, Sorel M (2016). Prefrontal tDCS decreases pain in patients with multiple sclerosis. Front Neurosci.

[261] Andrade SM, de Brito Aranha REL, de Oliveira EA, de Mendonca C, Martins WKN, Alves NT, Fernandez-Calvo B (2017). Transcranial direct current stimulation over the primary motor vs prefrontal cortex in refractory chronic migraine: a pilot randomized controlled trial. J Neurol Sci.

[262] Kim YJ, Ku J, Kim HJ, Im DJ, Lee HS, Han KA, Kang YJ (2013). Randomized, sham controlled trial of transcranial direct current stimulation for painful diabetic polyneuropathy. Annals of rehabilitation medicine.

[263] Wang J, Wang Y, Hu Z, Li X (2014). Transcranial direct current stimulation of the dorsolateral prefrontal cortex increased pain empathy. Neuroscience.

[264] DosSantos MF, Martikainen IK, Nascimento TD, Love TM, DeBoer MD, Schambra HM, Bikson M, Zubieta JK (2014). Building up analgesia in humans via the endogenous mu-opioid system by combining placebo and active tDCS: a preliminary report. PLoS One.

[265] Morgan V, Pickens D, Gautam S, Kessler R, Mertz H (2005). Amitriptyline reduces rectal pain related activation of the anterior cingulate cortex in patients with irritable bowel syndrome. Gut.

[266] Stratinaki M, Varidaki A, Mitsi V, Ghose S, Magida J, Dias C, Russo SJ, Vialou V (2013). Regulator of G protein signaling 4 [corrected] is a crucial modulator of antidepressant drug action in depression and neuropathic pain models. Proc Natl Acad Sci U S A.

[267] Schmidt-Wilcke T, Ichesco E, Hampson JP, Kairys A, Peltier S, Harte S, Clauw DJ, Harris RE (2014). Resting state connectivity correlates with drug and placebo response in fibromyalgia patients. Neuroimage Clin.

[268] Burke NN, Finn DP, Roche M (2015). Chronic administration of amitriptyline differentially alters neuropathic pain-related behaviour in the presence and absence of a depressive-like phenotype. Behav Brain Res.

[269] Benade V, Nirogi R, Bhyrapuneni G, Daripelli S, Ayyanki G, Irappanavar S, Ponnamaneni R, Manoharan A (2017). Mechanistic evaluation of tapentadol in reducing the pain perception using in-vivo brain and spinal cord microdialysis in rats. Eur J Pharmacol.

[270] Lee H, Im J, Won H, Nam W, Kim YO, Lee SW, Lee S, Cho IH (2017). Effects of tianeptine on symptoms of fibromyalgia via BDNF signaling in a fibromyalgia animal model. Korean J Physiol Pharmacol.

[271] Lee LH, Tan CH, Shui G, Wenk MR, Ong WY (2012). Role of prefrontal cortical calcium independent phospholipase A(2) in antidepressant-like effect of maprotiline. Int J Neuropsychopharmacol.

[272] Chew WS, Shalini SM, Torta F, Wenk MR, Stohler C, Yeo JF, Herr DR, Ong WY (2017). Role of prefrontal cortical calcium-independent phospholipase A2 in antinociceptive effect of the norepinephrine reuptake inhibitor antidepresssant maprotiline. Neuroscience.

[273] Li J, Zhang JH, Yi T, Tang WJ, Wang SW, Dong JC (2014). Acupuncture treatment of chronic low back pain reverses an abnormal brain default mode network in correlation with clinical pain relief. Acupunct Med.

[274] Maeda Y, Kettner N, Lee J, Kim J, Cina S, Malatesta C, Gerber J, McManus C (2013). Acupuncture-evoked response in somatosensory and prefrontal cortices predicts immediate pain reduction in carpal tunnel syndrome. Evid Based Complement Alternat Med.

[275] Dougherty DD, Kong J, Webb M, Bonab AA, Fischman AJ, Gollub RL (2008). A combined [11C]diprenorphine PET study and fMRI study of acupuncture analgesia. Behav Brain Res.

[276] Choi JC, Kim J, Kang E, Lee JM, Cha J, Kim YJ, Lee HG, Choi JH (2016). Brain mechanisms of pain relief by transcutaneous electrical nerve stimulation: a functional magnetic resonance imaging study. Eur J Pain.

[277] Seminowicz DA, Shpaner M, Keaser ML, Krauthamer GM, Mantegna J, Dumas JA, Newhouse PA, Filippi CG (2013). Cognitive-behavioral therapy increases prefrontal cortex gray matter in patients with chronic pain. J Pain.

[278] McLoughlin MJ, Stegner AJ, Cook DB (2011). The relationship between physical activity and brain responses to pain in fibromyalgia. J Pain.

[279] Younger J, Aron A, Parke S, Chatterjee N, Mackey S (2010). Viewing pictures of a romantic partner reduces experimental pain: involvement of neural reward systems. PLoS One.

[280] Bingel U, Colloca L, Vase L (2011). Mechanisms and clinical implications of the placebo effect: is there a potential for the elderly? A mini-review. Gerontology.

[281] Wager TD, Rilling JK, Smith EE, Sokolik A, Casey KL, Davidson RJ, Kosslyn SM, Rose RM (2004). Placebo-induced changes in FMRI in the anticipation and experience of pain. Science.

[282] Hashmi JA, Baria AT, Baliki MN, Huang L, Schnitzer TJ, Apkarian AV (2012). Brain networks predicting placebo analgesia in a clinical trial for chronic back pain. Pain.

[283] Lu HC, Hsieh JC, Lu CL, Niddam DM, Wu YT, Yeh TC, Cheng CM, Chang FY (2010). Neuronal correlates in the modulation of placebo analgesia in experimentally-induced esophageal pain: a 3T-fMRI study. Pain.

[284] Krummenacher P, Candia V, Folkers G, Schedlowski M, Schonbachler G (2010). Prefrontal cortex modulates placebo analgesia. Pain.

[285] Atlas LY, Wager TD (2014). A meta-analysis of brain mechanisms of placebo analgesia: consistent findings and unanswered questions. Handb Exp Pharmacol.

[286] Sevel LS, O'Shea AM, Letzen JE, Craggs JG, Price DD, Robinson ME (2015). Effective connectivity predicts future placebo analgesic response: a dynamic causal modeling study of pain processing in healthy controls. Neuroimage.

[287] Sevel LS, Craggs JG, Price DD, Staud R, Robinson ME (2015). Placebo analgesia enhances descending pain-related effective connectivity: a dynamic causal modeling study of endogenous pain modulation. J Pain.

[288] Tinnermann A, Geuter S, Sprenger C, Finsterbusch J, Buchel C (2017). Interactions between brain and spinal cord mediate value effects in nocebo hyperalgesia. Science.

[289] Ritter C, Hebart MN, Wolbers T, Bingel U (2014). Representation of spatial information in key areas of the descending pain modulatory system. J Neurosci.

[290] Schenk LA, Sprenger C, Onat S, Colloca L, Buchel C (2017). Suppression of striatal prediction errors by the prefrontal cortex in placebo hypoalgesia. J Neurosci.

[291] Liu J, Mu J, Liu Q, Dun W, Zhang M, Tian J (2017). Brain structural properties predict psychologically mediated hypoalgesia in an 8-week sham acupuncture treatment for migraine. Hum Brain Mapp.

[292] Wager TD, Scott DJ, Zubieta JK (2007). Placebo effects on human mu-opioid activity during pain. Proceedings of the National Academy of Sciences of the United States of America.

[293] Scott DJ, Stohler CS, Egnatuk CM, Wang H, Koeppe RA, Zubieta JK (2008). Placebo and nocebo effects are defined by opposite opioid and dopaminergic responses. Archives of general psychiatry.

[294] Zubieta JK, Stohler CS (2009). Neurobiological mechanisms of placebo responses. Annals of the New York Academy of Sciences.

[295] Zubieta JK, Bueller JA, Jackson LR, Scott DJ, Xu Y, Koeppe RA, Nichols TE, Stohler CS (2005). Placebo effects mediated by endogenous opioid activity on mu-opioid receptors. J Neurosci.

[296] Dobrila-Dintinjana R, Nacinovic-Duletic A (2011). Placebo in the treatment of pain. Collegium antropologicum.

[297] Lidstone SC, Stoessl AJ (2007). Understanding the placebo effect: contributions from neuroimaging. Molecular imaging and biology : MIB : the official publication of the Academy of Molecular Imaging.

[298] Bar KJ, Wagner G, Koschke M, Boettger S, Boettger MK, Schlosser R, Sauer H (2007). Increased prefrontal activation during pain perception in major depression. Biol Psychiatry.

[299] Giesecke T, Gracely RH, Williams DA, Geisser ME, Petzke FW, Clauw DJ (2005). The relationship between depression, clinical pain, and experimental pain in a chronic pain cohort. Arthritis Rheum.

[300] Berna C, Leknes S, Holmes EA, Edwards RR, Goodwin GM, Tracey I (2010). Induction of depressed mood disrupts emotion regulation neurocircuitry and enhances pain unpleasantness. Biol Psychiatry.

[301] Gustin SM, Peck CC, Macey PM, Murray GM, Henderson LA (2013). Unraveling the effects of plasticity and pain on personality. J Pain.

[302] Schweinhardt P, Kalk N, Wartolowska K, Chessell I, Wordsworth P, Tracey I (2008). Investigation into the neural correlates of emotional augmentation of clinical pain. NeuroImage.

[303] Qi WJ, Wang W, Wang N, Wang JY, Luo F (2013). Depressive-like history alters persistent pain behavior in rats: opposite contribution of frontal cortex and amygdala implied. Psych J.

[304] Xie ZM, Wang XM, Xu N, Wang J, Pan W, Tang XH, Zhou ZQ, Hashimoto K (2017). Alterations in the inflammatory cytokines and brain-derived neurotrophic factor contribute to depression-like phenotype after spared nerve injury: improvement by ketamine. Sci Rep.

[305] Descalzi Giannina, Mitsi Vasiliki, Purushothaman Immanuel, Gaspari Sevasti, Avrampou Kleopatra, Loh Yong-Hwee Eddie, Shen Li, Zachariou Venetia (2017). Neuropathic pain promotes adaptive changes in gene expression in brain networks involved in stress and depression. Science Signaling.

[306] Lee DH, Lee KJ, Cho KI, Noh EC, Jang JH, Kim YC, Kang DH (2015). Brain alterations and neurocognitive dysfunction in patients with complex regional pain syndrome. J Pain.

[307] Wiech K, Edwards R, Moseley GL, Berna C, Ploner M, Tracey I (2014). Dissociable neural mechanisms underlying the modulation of pain and anxiety? An FMRI pilot study. PLoS One.

[308] Qi J, Chen C, Lu YC, Zhang T, Xu H, Cui YY, Chen YZ, Wang W (2014). Activation of extracellular signal-regulated kinase1/2 in the medial prefrontal cortex contributes to stress-induced hyperalgesia. Mol Neurobiol.

[309] Tracey I (2007). Neuroimaging of pain mechanisms. Current opinion in supportive and palliative care.

[310] Seminowicz DA, Mikulis DJ, Davis KD (2004). Cognitive modulation of pain-related brain responses depends on behavioral strategy. Pain.

[311] Kunz M, Mylius V, Schepelmann K, Lautenbacher S (2015). Loss in executive functioning best explains changes in pain responsiveness in patients with dementia-related cognitive decline. Behav Neurol.

[312] Mao CP, Zhang QL, Bao FX, Liao X, Yang XL, Zhang M (2014). Decreased activation of cingulo-frontal-parietal cognitive/attention network during an attention-demanding task in patients with chronic low back pain. Neuroradiology.

[313] Luerding R, Weigand T, Bogdahn U, Schmidt-Wilcke T (2008). Working memory performance is correlated with local brain morphology in the medial frontal and anterior cingulate cortex in fibromyalgia patients: structural correlates of pain-cognition interaction. Brain.

[314] Ren X, Lu J, Liu X, Shen C, Zhang X, Ma X, Sun J, Sun G (2017). Decreased prefrontal brain activation during verbal fluency task in patients with somatoform pain disorder: an exploratory multi-channel near-infrared spectroscopy study. Prog Neuropsychopharmacol Biol Psychiatry.

[315] Gosselin N, Chen JK, Bottari C, Petrides M, Jubault T, Tinawi S, de Guise E, Ptito A (2012). The influence of pain on cerebral functioning after mild traumatic brain injury. J Neurotrauma.

[316] Cheng JC, Bosma RL, Hemington KS, Kucyi A, Lindquist MA, Davis KD (2017). Slow-5 dynamic functional connectivity reflects the capacity to sustain cognitive performance during pain. Neuroimage.

[317] Weissman-Fogel I, Moayedi M, Tenenbaum HC, Goldberg MB, Freeman BV, Davis KD (2011). Abnormal cortical activity in patients with temporomandibular disorder evoked by cognitive and emotional tasks. Pain.

[318] Attal N, Masselin-Dubois A, Martinez V, Jayr C, Albi A, Fermanian J, Bouhassira D, Baudic S (2014). Does cognitive functioning predict chronic pain? Results from a prospective surgical cohort. Brain : a journal of neurology.

[319] Schmidt G, Alvarenga R, Manhaes A, Schmidt S (2017). Attentional performance may help to identify duloxetine responders in chronic pain fibromyalgia patients. Eur J Pain.

[320] Ceko M, Gracely JL, Fitzcharles MA, Seminowicz DA, Schweinhardt P, Bushnell MC (2015). Is a responsive default mode network required for successful working memory task performance?. J Neurosci.

[321] Jahn A, Nee DE, Alexander WH, Brown JW (2016). Distinct regions within medial prefrontal cortex process pain and cognition. J Neurosci.

[322] Kragel PA, Kano M, Van Oudenhove L, Ly HG, Dupont P, Rubio A, Delon-Martin C, Bonaz BL (2018). Generalizable representations of pain, cognitive control, and negative emotion in medial frontal cortex. Nat Neurosci.

[323] Pais-Vieira M, Aguiar P, Lima D, Galhardo V (2012). Inflammatory pain disrupts the orbitofrontal neuronal activity and risk-assessment performance in a rodent decision-making task. Pain.

[324] Cardoso-Cruz H, Lima D, Galhardo V (2013). Impaired spatial memory performance in a rat model of neuropathic pain is associated with reduced hippocampus-prefrontal cortex connectivity. J Neurosci.

[325] Cowen Stephen L., Phelps Caroline E., Navratilova Edita, McKinzie David L., Okun Alec, Husain Omar, Gleason Scott D., Witkin Jeffrey M., Porreca Frank (2018). Chronic pain impairs cognitive flexibility and engages novel learning strategies in rats. PAIN.

[326] Zhang X, Xin X, Dong Y, Zhang Y, Yu B, Mao J, Xie Z (2013). Surgical incision-induced nociception causes cognitive impairment and reduction in synaptic NMDA receptor 2B in mice. J Neurosci.

[327] D'Aniello A, Luongo L, Romano R, Iannotta M, Marabese I, Boccella S, Belardo C, de Novellis V (2017). d-Aspartic acid ameliorates painful and neuropsychiatric changes and reduces beta-amyloid Abeta1-42 peptide in a long lasting model of neuropathic pain. Neurosci Lett.

[328] Ruscheweyh R, Deppe M, Lohmann H, Stehling C, Floel A, Ringelstein EB, Knecht S (2011). Pain is associated with regional grey matter reduction in the general population. Pain.

[329] Seminowicz DA, Wideman TH, Naso L, Hatami-Khoroushahi Z, Fallatah S, Ware MA, Jarzem P, Bushnell MC (2011). Effective treatment of chronic low back pain in humans reverses abnormal brain anatomy and function. J Neurosci.

[330] Ceko M, Shir Y, Ouellet JA, Ware MA, Stone LS, Seminowicz DA (2015). Partial recovery of abnormal insula and dorsolateral prefrontal connectivity to cognitive networks in chronic low back pain after treatment. Hum Brain Mapp.

[331] Rodriguez-Raecke R, Niemeier A, Ihle K, Ruether W, May A (2009). Brain gray matter decrease in chronic pain is the consequence and not the cause of pain. J Neurosci.

[332] Rodriguez-Raecke R, Niemeier A, Ihle K, Ruether W, May A (2013). Structural brain changes in chronic pain reflect probably neither damage nor atrophy. PLoS One.

[333] Chen X, Spaeth RB, Retzepi K, Ott D, Kong J (2014). Acupuncture modulates cortical thickness and functional connectivity in knee osteoarthritis patients. Sci Rep.

[334] Erpelding N, Simons L, Lebel A, Serrano P, Pielech M, Prabhu S, Becerra L, Borsook D (2016). Rapid treatment-induced brain changes in pediatric CRPS. Brain Struct Funct.

[335] Seminowicz DA, Ceko M (2015). Can we exploit cognitive brain networks to treat chronic pain?. Pain Manag.

